# Supplementary material for: 5-Aryl-2-(3,5-dialkyl-4-hydroxyphenyl)-4,4-dimethyl-4H-imidazole 3-Oxides and Their Redox Species: How Antioxidant Activity of 1-Hydroxy-2,5-dihydro-1H-imidazoles Correlates with the Stability of Hybrid Phenoxyl–Nitroxides
Source: Molecules. 2020 Jul 8;25(14):3118. doi: 10.3390/molecules25143118 (PMC7396990; doi:10.3390/molecules25143118)
Supplement: Supplementary file 1 [file molecules-25-03118-s001.pdf]

Supplementary Materials

for the paper

"5-Aryl-2-(3,5-dialkyl-4-hydroxyphenyl)-4,4-dimethyl-4*H*-imidazole 3-Oxides and Their Redox Species: How Antioxidant Activity of 1-Hydroxy-2,5-dihydro-1*H*-imidazoles Correlates with the Stability of Hybrid Phenoxyl-Nitroxides"

S.A. Amitina, E.V. Zaytseva, N.A. Dmitrieva, A.V. Lomanovich, N. V. Kandalintseva, Y.A. Ten, I.A. Artamonov, A.F. Markov, D.G. Mazhukin

Content

Synthesis of 2-hydroxylamino ketone hydrochloride **25c×HCl**	S1
Physicochemical, analytical and spectral data for compounds **23a,c,d**; **20a-s** and **21a-s**	S3
Description of chain reactions in the hydrocarbon oxidation process	S12
ESR spectra of diluted and oxygen-fee toluene solutions of HPNs **22a-o**	S13
Molecular geometry and *hfs* constants of HPNs **22a-e,j,o** calculated at UB3LYP/6-31G(d)	S21

Synthesis

Synthesis of 1-(4-bromophenyl)-2-(hydroxyamino)-2-methylpropan-1-one hydrochloride (**25c×HCl**) was carried out in four steps: 1) *Friedel-Crafts* acylation of bromobenzene by isobutyryl chloride [1]; 2) bromination of the obtained *para*-bromo-*iso*butyrophenone; 3) treatment of bromoketone **26c** with excess of hydroxylamine, followed by 4) acid catalized hydrolysis of 2-hydroxylaminooxime **28c**, according to Scheme S1.

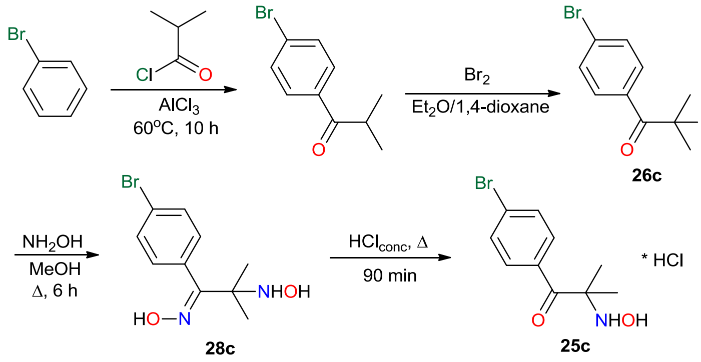

Scheme S1. Preparation of 2-hydroxyamino ketone hydrochloride **25c×HCl**.

1-(4-Bromophenyl)-2-methylpropan-1-one. Freshly sublimed $AlCl_3$ (53.5 g, 0.4 mol) was added to the vigorously stirred warm (+30°C) mixture of bromobenzene (127 mL, 1.2 mol) and *iso*-butyryl chloride (50 mL, 0.475 mol) in five portions during 20 min, so as the temperature of the reaction mixture did not exceed +45°C. The slurry was warmed to 55÷60°C and stirring was continued during 11 h. After cooling to ambient temperature, the reaction mass was poured into the mixture of concentrated HCl with ice (1/1, 300 mL). An organic layer was extracted by chloroform (3×50 mL),

washed with water (30 mL) and saturated aq $NaHCO_3$ (100 mL), dried over anhydrous Na_2SO_4 and evaporated. The residue was distilled in vacuo and three fractions were collected consequently: a) unreacted bromobenzene boiling at 40÷85°C, b) mixture of dibromobenzene and *iso*-butyrophenone boiling at 85÷115°C and c) the purposed product with b.p. 115÷134°C (8 mm). The latter fraction was redistilled in vacuo using short column, the yield 67% of 1-(4-bromophenyl)-2-methylpropan-1-one in terms of the catalyst was achieved.

Slightly yellowish oily liquid, total mass 60.6 g, b. p. 127÷132°C (8 mm). ^{1}H NMR spectrum of ketone was identical to that published in literature [2,3].

2-Bromo-1-(4-bromophenyl)-2-methylpropan-1-one (26c). Bromine (31.5 mL, 0.6 mol) was added dropwise to a stirred solution of 1-(4-bromophenyl)-2-methylpropan-1-one (136g, 0.6 mol) in a mixture of 300 ml of diethyl ether and 20 ml of 1,4-dioxane in such a rate that the next drop was added to the colorless solution. The mixture was stirred during 90 min and then cooled in an ice bath. Water (100 mL) and sodium bicarbonate (57 g, 0.6 mol) in small portions were added consequently to the solution. The organic layer was separated, the water layer was extracted with Et_2O (2×20 mL), combined ether extract was dried over $MgSO_4$, filtered through the thin layer of alumina and the solvent was evaporated giving bromoketone **26c**, which was used for the next step without further purification.

Pale-yellow viscous liquid (***caution – lacrimator!***), total mass 196.4 g (contains ~7% 1,4-dioxane as an admixture). ^{1}H NMR spectrum of a sample was identical to that published in literature [4].

(*E*)-1-(4-Bromophenyl)-2-(hydroxyamino)-2-methylpropan-1-one oxime (28c). A suspension of 104.18 g (1.50 mol) of hydroxylamine hydrochloride in 900 mL of methanol was heated until dissolution, then rapidly cooled and neutralized thoroughly in a cold bath with a solution of 50.50 g (1.26 mol) of NaOH in 75 mL of water. The precipitate of NaCl was filtered off and filtrate was mixed with a solution of 98.00 g (~0.30 mol) of 2-bromo-1-(4-bromophenyl)-2-methylpropan-1-one **26c** in 200 mL of MeOH followed by heating and refluxing of the mixture for 15 h. The methanol was removed under vacuum, the semi crystalline residue was triturated with 300 mL of water, and the precipitate of 2-hydroxylaminooxime **28c** was filtered, thoroughly washed with cold water and dried in air to constant weight.

Colorless powder, isolated yield 62.15 g (76%), mp 170–172 °C (MeOH). Elemental analysis: found: C, 44.16; H, 4.83; Br, 29.36; N, 10.09; calcd. for $C_{10}H_{13}BrN_2O_2$: C, 43.97; H, 4.80; Br, 29.26; N, 10.26%. ^{1}H NMR (500 MHz, DMSO-d_6), δ, ppm (*J*, Hz): 1.11 (6H, s, $(CH_3)_2$); 5.17 (1H, br.s, N*H*OH); 7.13 (2H, *AA′BB′*, *J* = 8.4, H_A: H-3,5 ArBr); 7.37 (1H, s, NHO*H*); 7.56 (2H, *AA′BB′*, *J* = 8.4, H_B: H-2,6 ArBr); 10.64 (1H, s, C=NOH). ^{13}C NMR (125 MHz, DMSO-d_6), δ, ppm: 23.6 (C-CH3); 60.9 (*C*-CH3); 121.1 (C-Br); 130.7, 130.9 (CH Ar); 133.2 (C-1 Ar); 160.5 (C=N).

1-(4-Bromophenyl)-2-(hydroxyamino)-2-methylpropan-1-one hydrochloride (25c×HCl). A mixture of 2-hydroxylamino oxime **28c** (13.60 g, 50 mmol) and 40 mL of conc. hydrochloric acid (ϱ 1.18 g/mL) was refluxed during 50 min, then it was cooled until rt and kept for 16 h. The formed precipitate was filtered off, washed with 10 mL of hydrochloric acid and transferred into a flask filled with 60 mL of hydrochloric acid. The mixture was heated again until the complete crystals dissolution (10-15 min). After cooling and refrigeration of the mixture at 0 °C for 24 h, the formed precipitate of **25c×HCl** was filtered off, washed with cold acetonitrile (3×6 mL) and dried in air to constant weight.

Colorless crystals, isolated yield 12.80 g (84%), mp 168–172 °C (MeCN / EtOH, 3:1). Elemental analysis: found: C, 40.84; H, 4.41; Br, 27.00; Cl, 11.96; N, 4.94; calcd. for $C_{10}H_{13}BrClNO_2$: C 40.77; H 4.45; Br 27.13; Cl 12.04; N 4.75 %. ^{1}H NMR (500 MHz, DMSO-d_6), δ, ppm (*J*, Hz): 1.64 (6H, s, $(CH_3)_2$); 7.71 (2H, *AA′BB′*, *J* = 8.7, H_A: H-3,5 ArBr); 7.82 (2H, *AA′BB′*, *J* = 8.7, H_B: H-2,6 ArBr); 10.92 (1H, br.s, N^+H_2). ^{13}C NMR (125 MHz, DMSO-d_6), δ, ppm: 20.5 (C-*C*H3); 69.1 (*C*-CH3); 127.8 (C-Br); 131.0, 132.2 (CH Ar); 132.9 (C-1 Ar); 198.2 (C=O).

Synthesis of 1-(4-fluorophenyl)-2-(hydroxyamino)-2-methylpropan-1-one hydrochloride (**25b×HCl**) from fluorobenzene was performed in a similar way.

Physicochemical, analytical and spectral data for compounds **23a,c-e**; **20a-s** and **21a-s**

4-Hydroxy-3,5-dimethylbenzaldehyde (23a). Colorless crystals, yield before crystallization 95%, m. p. 114-115°C (water-ethanol) (lit. m. p. 113-115°C [5]).

4-Hydroxy-3,5-diisopropylbenzaldehyde (23c). Colorless crystals, yield before crystallization 85%, m. p. 107-108°C (*i*-PrOH)(lit. m. p. 101-103°C (*i*-PrOH)[6]), ^{1}H and ^{13}C NMR spectra were similar to those presented in literature [7].

3,5-Dicyclohexyl-4-hydroxybenzaldehyde (23d). Colorless crystals, yield after crystallization 94%, m. p. 175-176°C (benzene, decomp.)(lit. m.p. 175-176°C (PhH / petroleum ether) [8]). Elemental analysis: found: C, 79.60; H, 9.24; calcd. for $C_{19}H_{26}O_2$: C, 79.68; H, 9.15%. UV (EtOH), λ_{max}nm, (lg ε): 231 (4.26), 294 (4.18). ^{1}H NMR (500 MHz, $CDCl_3$), δ, ppm (*J*, Hz): 1.19-1.32 (2H, m, 2C_6H_{11}); 1.35-1.49 (8H, m, 2C_6H_{11}); 1.70-1.79 (2H, m, 2C_6H_{11}); 1.80-1.94 (8H, m, 2C_6H_{11}); 2.72-2.82 (2H, m, Ar-C*H*-(CH_2)$_5$); 5.86 (1H, br.s, OH); 7.58 (2H, s, H-2,6); 9.82 (1H, s, CHO). ^{13}C NMR (125 MHz, $CDCl_3$), δ, ppm: 26.0, 26.7, 32.9 (3 CH_2); 37.3 (CH-C=C); 126.6 (CH Ar); 129.5 (C Ar); 133.5 (C Ar); 155.9 (C-OH Ar); 191.8 (CHO).

3,5-Di-*tert*-butyl-4-hydroxybenzaldehyde (23e). Colorless crystals, yield after crystallization 80%, m. p. 190°C (PhMe)(lit. m. p. 189 °C (PhMe)), ^{1}H NMR spectrum was similar to that presented in literature [9].

2-(4-Hydroxy-3,5-dimethylphenyl)-5,5-dimethyl-4-phenyl-2,5-dihydro-1*H*-imidazol-1-ol (20a). Colorless powder, yield 1.62 g (87%), m.p. 198-202 °C (EtOH, dec.). Elemental analysis: found: C, 73.52; H, 7.24; N, 9.02; calcd. for $C_{19}H_{22}N_2O_2$: C, 73.52; H, 7.14; N, 9.03%. IR-spectrum, ν, cm^{-1}, (KBr): 3248 (OH), 2979, 2935 (CH), 1602 (C=N), 1488, 1215, 1157. ^{1}H NMR (300 MHz, DMSO-d_6), δ, ppm (*J*, Hz): 1.37 (3H, s, 5-CH_3); 1.39 (3H, s, 5-CH_3); 2.19 (6H, s, 3,5-CH_3 Ar); 5.31 (1H, s, H-2); 7.00 (2H, s, H-2,6 Ar); 7.42-7.53 (3H, m, Ph); 7.75 (1H, s, N-OH); 7.81-7.87 (2H, m, Ph); 8.14 (1H, s, Ar-OH). ^{13}C NMR (75 MHz, DMSO-d_6), δ, ppm: 16.5 (5-CH_3); 16.7 (Ar-CH_3); 25.4 (5-CH_3); 70.4 (C-5); 89.3 (C-2); 123.5 (CH_3-*C* Ar); 127.4; 127.9; 128.5; 130.4 (*C*H (Ar + Ph)); 131.3; 133.0 (*C*(Ar)-Het and *C*(Ph)-Het); 152.7 (C-OH); 175.4 (C-4).

2-(3-Cyclohexyl-4-hydroxy-5-methylphenyl)-5,5-dimethyl-4-phenyl-2,5-dihydro-1*H*-imidazol-1-ol (20b). Colorless powder, yield 1.97 g (87%), m.p. 190-192 °C (MeOH, dec.). Elemental analysis: found: C, 75.85; H, 7.97; N, 7.00; calcd. for $C_{24}H_{30}N_2O_2$: C, 76.16; H, 7.99; N, 7.40%. IR-spectrum, ν, cm^{-1}, (KBr): 3234 (OH), 2928, 2851 (CH), 1595 (C=N), 1446, 1221, 1158. ^{1}H NMR (300 MHz, DMSO-d_6), δ, ppm (*J*, Hz): 1.17-1.48 (5H, m, C_6H_{11}); 1.38 (3H, s, 5-CH_3); 1.40 (3H, s, 5-CH_3); 1.66-1.85 (5H, m, C_6H_{11}); 2.21 (3H, s, 5-CH_3 Ar); 2.89-3.01 (1H, m, Ar-C*H*-(CH_2)$_5$); 5.35 (1H, s, H-2); 7.01 (1H, s, H-6 Ar); 7.07 (1H, s, H-2 Ar); 7.42-7.54 (3H, m, Ph); 7.79 (1H, s, N-OH); 7.82-7.88 (2H, m, Ph); 8.07 (1H, s, Ar-OH). ^{13}C NMR (75 MHz, DMSO-d_6), δ, ppm: 16.5 (5-CH_3); 17.0 (Ar-CH_3); 25.4 (5-CH_3); 25.9; 26.7; 33.0 (3 CH_2); 36.5 (CH in C_6H_{11}); 70.5 (C-5); 89.5 (C-2); 123.8 (CH_3-*C* Ar); 123.9; 127.4; 128.5; 130.4 (CH (Ar + Ph)); 131.5; 133.0; 133.5 (*C*(Ar)-Het, *C*(Ar)-C_6H_{11} and *C*(Ph)-Het); 151.6 (C-OH); 175.5 (C-4).

2-(4-Hydroxy-3,5-diisopropylphenyl)-5,5-dimethyl-4-phenyl-2,5-dihydro-1*H*-imidazol-1-ol (20c). Colorless plates, yield 1.65 g (75%), m.p. 209-210 °C (MeOH, dec.). Elemental analysis: found: C, 75.31; H, 8.22; N, 7.63; calcd. for $C_{23}H_{30}N_2O_2$: C, 75.37; H, 8.25; N, 7.64%. IR-spectrum, ν, cm^{-1}, (KBr): 3282 (OH), 2964, 2871 (CH), 1591 (C=N), 1570, 1461, 1282. ^{1}H NMR (300 MHz, DMSO-d_6), δ, ppm (*J*, Hz): 1.18 (12H, d, *J* = 6.9, C*H*$_3$-CH-C*H*$_3$); 1.40 (6H, s, 5-CH_3); 3.33 (2H, sept., *J* = 6.9, CH_3-C*H*-CH_3); 5.38 (1H, s, H-2); 7.09 (2H, s, H-2,6 Ar); 7.43-7.53 (3H, m, Ph); 7.81 (1H, s, N-OH); 7.82-7.88 (2H, m, Ph); 8.00 (1H, s, Ar-OH). ^{13}C NMR (75 MHz, DMSO-d_6), δ, ppm: 16.6 (5-CH_3); 23.1 (Ar-CH-CH_3); 25.4 (5-CH_3); 26.3 (Ar-CH-CH_3); 70.6 (C-5); 89.7 (C-2); 122.7 (CH Ar), 127.4; 128.6; 130.4 (CH Ph); 131.9; 133.1 (*C*(Ar)-Het and *C*(Ph)-Het); 134.5 (*C*(Ar)-CH-CH_3); 150.3 (C-OH); 175.5 (C-4).

2-(3,5-Dicyclohexyl-4-hydroxyphenyl)-5,5-dimethyl-4-phenyl-2,5-dihydro-1*H*-imidazol-1-ol (20d). Faint yellow powder, yield 2.45 g (91%), m.p. 223-225 °C (MeCN, dec.). Elemental analysis: found: C, 78.01; H, 8.54; N, 6.34; calcd. for $C_{29}H_{38}N_2O_2$: C, 77.99; H, 8.58; N, 6.27%. IR-spectrum, ν, cm^{-1}, (KBr): 3285 (OH), 2930, 2850 (CH), 1592 (C=N), 1566, 1448. ^{1}H NMR (400 MHz, DMSO-d_6), δ, ppm (*J*, Hz): 1.29-1.53 (10H, m, C_6H_{11}); 1.37 (3H, s, 5-CH_3); 1.39 (3H, s, 5-CH_3); 1.65-1.79 (10H, m, C_6H_{11}); 2.91-2.98 (2H, m, Ar-C*H*-$(CH_2)_5$); 5.34 (1H, s, H-2); 7.03 (2H, s, H-2,6 Ar); 7.44-7.52 (3H, m, Ph); 7.79 (1H, s, N-OH); 7.83-7.87 (2H, m, Ph); 7.93 (1H, s, Ar-OH).

2-(3,5-Di-*tert*-butyl-4-hydroxyphenyl)-5,5-dimethyl-4-phenyl-2,5-dihydro-1*H*-imidazol-1-ol (20e). Colorless powder, yield 1.85 g (78%), m.p. 200-202 °C (EtOH, dec.). Elemental analysis: found: C, 76.17; H, 8.58; N, 7.14; calcd. for $C_{25}H_{34}N_2O_2$: C, 76.10; H, 8.69; N, 7.10%. IR-spectrum, ν, cm^{-1}, (KBr): 3435 (OH), 2964 (CH), 1603 (C=N), 1575, 1435, 1365, 1239. ^{1}H NMR (300 MHz, DMSO-d_6), δ, ppm (*J*, Hz): 1.39 (3H, s, 5-CH_3); 1.41 (21H, s, 5-CH_3 and 2 *t*-Bu); 5.37 (1H, s, H-2); 6.89 (1H, s, Ar-OH); 7.21 (2H, s, H-2,6 Ar); 7.41-7.54 (3H, m, Ph); 7.79-7.87 (2H, m, Ph); 7.81 (1H, s, N-OH). ^{13}C NMR (75 MHz, DMSO-d_6), δ, ppm: 16.6 (5-CH_3); 25.5 (5-CH_3); 30.4 (C($CH_3)_3$); 34.5 (*C*($CH_3)_3$); 70.5 (C-5); 89.8 (C-2); 124.2 (CH Ar); 127.4; 128.6; 130.5 (CH Ph)); 131.8; 133.1 (*C*(Ar)-Het and *C*(Ph)-Het); 138.5 (*C*(Ar)-*t*-Bu); 153.5 (C-OH); 175.7 (C-4).

4-(4-Fluorophenyl)-2-(4-hydroxy-3,5-dimethylphenyl)-5,5-dimethyl-2,5-dihydro-1*H*-imidazol-1-ol (20f). Colorless powder, yield 1.71 g (87%), m.p. 198-202 °C (80% EtOH, dec.). Elemental analysis: found: C, 69.23; H, 6.77; F, 5.95; N, 8.21; calcd. for $C_{19}H_{21}FN_2O_2$: C, 69.49; H, 6.45; F, 5.79; N, 8.53%. IR-spectrum, ν, cm^{-1}, (KBr): 3469, 3241 (OH), 2939 (CH), 1606 (C=N), 1511, 1222, 1155. ^{1}H NMR (400 MHz, DMSO-d_6), δ, ppm (*J*, Hz): 1.35 (3H, s, 5-CH_3); 1.38 (3H, s, 5-CH_3); 2.18 (6H, s, 3,5-CH_3 Ar); 5.29 (1H, s, H-2); 6.99 (2H, s, H-2,6 ArOH); 7.28 (2H, ddd, $^3J_{HF}$ = 8.0, ^3J = 7.5, ^4J = 1.5, H-3′,5′ ArF); 7.76 (1H, s, N-OH); 7.90 (2H, ddd, ^3J = 7.5, $^4J_{HF}$ = 5.0, ^4J = 1.5, H-2′,6′ ArF); 8.14 (1H, s, Ar-OH).

2-(3-Cyclohexyl-4-hydroxy-5-methylphenyl)-4-(4-fluorophenyl)-5,5-dimethyl-2,5-dihydro-1*H*-imidazol-1-ol (20g). Light yellow microcrystals, yield 2.16 g (91%), m.p. 184-185 °C (MeOH, dec.). Elemental analysis: found: C, 73.02; H, 7.26; F, 4.86; N, 7.19; calcd. for $C_{24}H_{29}FN_2O_2$: C, 72.70; H, 7.37; F, 4.79; N, 7.07. IR-spectrum, ν, cm^{-1}, (KBr): 3257 (OH), 2926, 2852 (CH), 1606 (C=N), 1513, 1237, 1156. ^{1}H NMR (300 MHz, DMSO-d_6), δ, ppm (*J*, Hz): 1.18-1.45 (5H, m, C_6H_{11}); 1.36 (3H, s, 5-CH_3); 1.40 (3H, s, 5-CH_3); 1.67-1.87 (5H, m, C_6H_{11}); 2.20 (3H, s, 5-CH_3 Ar); 2.88-3.02 (1H, m, Ar-C*H*-$(CH_2)_5$); 5.33 (1H, s, 2-H); 6.99 (1H, d, ^4J = 1.5, H-6′ ArOH); 7.05 (1H, d, ^4J = 1.5, H-2′ ArOH); 7.28 (2H, ddd, $^3J_{HF}$ = 8.0, ^3J = 7.5, ^4J = 1.5, H-3′,5′ ArF); 7.79 (1H, s, N-OH); 7.91 (2H, ddd, ^3J = 7.5, $^4J_{HF}$ = 5.0, ^4J = 1.5, H-2′,6′ ArF); 8.02 (1H, s, Ar-OH). ^{13}C NMR (75 MHz, DMSO-d_6), δ, ppm: 16.2 (5-CH_3); 16.8 (Ar-CH_3); 25.1 (5-CH_3); 25.7; 26.5; 32.8; 32.9 (4 CH_2); 36.4 (CH in C_6H_{11}); 70.2 (C-5); 89.3 (C-2); 115.3 (d, $^2J_{CF}$ = 21.5, C-3′,5′ ArF); 123.6 (C-6′(2′) ArOH); 123.6 (Me-*C* ArOH); 127.2 (C-2′(6′) ArOH); 129.2 (d, $^4J_{CF}$ = 3.0, C-1′ ArF); 129.7 (d, $^3J_{CF}$ = 8.6, C-2′,6′ ArF); 131.3 (C-5′(1′) ArOH); 133.3 (C-1′(5′) ArOH); 151.4 (C-OH); 163.2 (d $^1J_{CF}$ = 247, C-4′ ArF); 174.1 (C-4).

4-(4-Fluorophenyl)-2-(4-hydroxy-3,5-diisopropylphenyl)-5,5-dimethyl-2,5-dihydro-1*H*-imidazol-1-ol (20h). Colorless powder, yield 1.95 g (85%), m.p. 175-177 °C (MeOH, dec.). Elemental analysis: found: C, 72.11; H, 7.55; F, 5.21; N, 7.35; calcd. for $C_{23}H_{29}FN_2O_2$: C, 71.85; H, 7.60; F, 4.94; N, 7.29%. IR-spectrum, ν, cm^{-1}, (KBr): 3283 (OH), 2964, 2870 (CH), 1606 (C=N), 1512, 1470, 1236, 1157. ^{1}H NMR (300 MHz, DMSO-d_6), δ, ppm (*J*, Hz): 1.17 (12H, d, *J* = 6.8, C*H*$_3$-CH-C*H*$_3$); 1.37 (3H, s, 5-CH_3); 1.39 (3H, s, 5-CH_3); 3.32 (2H, septet, *J* = 6.8, CH_3-C*H*-CH_3); 5.35 (1H, s, H-2); 7.06 (2H, s, H-2,6 ArOH); 7.29 (2H, ddd, $^3J_{HF}$ = 8.0, ^3J = 7.5, ^4J = 1.5, H-3′,5′ ArF); 7.81 (1H, s, N-OH); 7.91 (2H, ddd, ^3J = 7.5, $^4J_{HF}$ = 5.0, ^4J = 1.5, H-2′,6′ ArF); 7.99 (1H, s, Ar-OH).

2-(3,5-Dicyclohexyl-4-hydroxyphenyl)-4-(4-fluorophenyl)-5,5-dimethyl-2,5-dihydro-1*H*-imidazol-1-ol (20i). Faint yellow crystals, yield 2.28 g (82%), m.p. 199-203 °C (MeOH, dec.). Elemental analysis: found: C, 74.60; H, 7.84; F, 4.27; N, 6.10; calcd. for $C_{29}H_{37}FN_2O_2$: C, 74.97; H, 8.03; F, 4.09; N, 6.03%. IR-

spectrum, ν, cm⁻¹, (KBr): 3310 (OH), 2928, 2852 (CH), 1606 (C=N), 1513, 1465, 1237. ^{1}H NMR (300 MHz, DMSO-*d*$_6$), δ, ppm (*J*, Hz): 1.15-1.48 (10H, m, C_6H_{11}); 1.36 (3H, s, 5-CH_3); 1.40 (3H, s, 5-CH_3); 1.67-1.85 (10H, m, C_6H_{11}); 2.89-3.02 (2H, m, Ar-C*H*-(CH_2)$_5$); 5.34 (1H, s, H-2); 7.03 (2H, s, H-2,6 ArOH); 7.29 (2H, ddd, $^3J_{HF}$ = 8.0, ^3J = 7.5, ^4J = 1.5, H-3′,5′ ArF); 7.81 (1H, s, N-OH); 7.91 (2H, ddd, ^3J = 7.5, $^4J_{HF}$ = 5.0, ^4J = 1.5, H-2′,6′ ArF); 7.91 (1H, s, Ar-OH). ^{13}C NMR (75 MHz, DMSO-*d*$_6$), δ, ppm: 16.4 (5-CH_3); 25.4 (5-CH_3); 25.9; 26.6; 33.1; 33.2 (4 CH_2); 36.5 (CH in C_6H_{11}); 70.5 (C-5); 89.7 (C-2); 115.6 (d, $^2J_{CF}$ = 21.5, C-3′,5′ ArF); 123.2 (C-2′,6′ ArOH); 129.5 (d, $^4J_{CF}$ = 2.9, C-1′ ArF); 129.9 (d, $^3J_{CF}$ = 8.6, C-2′,6′ ArF); 131.7 (C-1′ ArOH); 133.8 (C-3′,5′ ArOH); 150.3 (C-OH); 163.4 (d, $^1J_{CF}$ = 247, C-4′ ArF); 174.4 (C-4).

2-(3,5-Di-*tert*-butyl-4-hydroxyphenyl)-4-(4-fluorophenyl)-5,5-dimethyl-2,5-dihydro-1*H*-imidazol-1-ol (20j). Colorless crystals, yield 1.88 g (76%), m.p. 185-190 °C (EtOH, dec.). Elemental analysis: found: C, 72.83; H, 7.95; F, 4.63; N, 6.44; calcd. for $C_{25}H_{33}FN_2O_2$: C, 72.79; H, 8.06; F, 4.61; N, 6.79%. IR-spectrum, ν, cm⁻¹, (KBr): 3620, 3419, 3264, (OH), 2959 (CH), 1608 (C=N), 1512, 1438, 1235. ^{1}H NMR (300 MHz, DMSO-*d*$_6$), δ, ppm (*J*, Hz): 1.37 (3H, s, 5-CH_3); 1.38 (3H, s, 5-CH_3); 1.41 (18H, s, *t*-Bu); 5.34 (1H, s, H-2); 6.88 (1H, s, Ar-OH); 7.19 (2H, s, H-2,6 ArOH); 7.30 (2H, ddd, $^3J_{HF}$ = 8.0, ^3J = 7.5, ^4J = 1.5, H-3′,5′ ArF); 7.81 (1H, s, N-OH); 7.91 (2H, ddd, ^3J = 7.5, $^4J_{HF}$ = 5.0, ^4J = 1.5, H-2′,6′ ArF). ^{13}C NMR (75 MHz, DMSO-*d*$_6$), δ, ppm: 16.4 (5-CH_3); 25.4 (5-CH_3); 30.4 (C(CH_3)$_3$); 34.5 (*C*(CH_3)$_3$); 70.4 (C-5); 89.7 (C-2); 115.6 (d, $^2J_{CF}$ = 21.5, C-3′,5′ ArF); 124.2 (C-2′,6′ ArOH); 129.4 (d, $^4J_{CF}$ = 3.0, C-1′ ArF); 129.8 (d, $^3J_{CF}$ = 8.6, C-2′,6′ ArF); 131.7 (C-1′ ArOH); 138.5 (C-3′,5′ ArOH); 153.5 (C-OH); 163.4 (d, $^1J_{CF}$ = 247, C-4′ ArF); 174.6 (C-4).

4-(4-Bromophenyl)-2-(4-hydroxy-3,5-dimethylphenyl)-5,5-dimethyl-2,5-dihydro-1*H*-imidazol-1-ol (20k). Colorless fine crystals, yield 2.28 g (98%), m.p. 204-205 °C (MeOH, dec.). Elemental analysis: found: C, 58.70; H, 5.28; Br, 20.70; N, 7.23; calcd. for $C_{19}H_{21}BrN_2O_2$: C, 58.62; H, 5.44; Br, 20.53; N, 7.20%. IR-spectrum, ν, cm⁻¹, (KBr): 3248 (OH), 2984, 2935 (CH), 1602 (C=N), 1489, 1210, 1157. ^{1}H NMR (400 MHz, DMSO-*d*$_6$), δ, ppm (*J*, Hz): 1.34 (3H, s, 5-CH_3); 1.37 (3H, s, 5-CH_3); 2.18 (6H, s, 3,5-CH_3 Ar); 5.29 (1H, s, H-2); 6.99 (2H, s, H-2,6 ArOH); 7.65 (2H, *AA′BB′*, *J* = 8.5, H_A: H-3′,5′ ArBr); 7.77 (2H, *AA′BB′*, *J* = 8.5, H_B: H-2′,6′ ArBr); 7.78 (1H, s, N-OH); 8.18 (1H, s, Ar-OH). ^{13}C NMR (100 MHz, DMSO-*d*$_6$), δ, ppm: 16.5 (5-CH_3); 16.8 (Ar-CH_3); 25.2 (5-CH_3); 70.4 (C-5); 89.3 (C-2); 123.5 (CH_3-*C* Ar); 124.2 (C-Br); 127.9; 129.6; 131.7 (CH ArOH and ArBr); 131.1; 132.0 (*C*(ArBr)-Het and *C*(ArOH)-Het); 152.8 (C-OH); 174.7 (C-4).

4-(4-Bromophenyl)-2-(3-cyclohexyl-4-hydroxy-5-methylphenyl)-5,5-dimethyl-2,5-dihydro-1*H*-imidazol-1-ol (20l). Colorless powder, yield 2.55 g (93%), m.p. 204-205 °C (MeOH, dec.). Elemental analysis: found: C, 63.35; H, 6.34; Br, 17.41; N, 6.30; calcd. for $C_{24}H_{29}BrN_2O_2$: C, 63.02; H, 6.39; Br, 17.47; N, 6.12%. IR-spectrum, ν, cm⁻¹, (KBr): 3262 (OH), 2974, 2929, 2851 (CH), 1612 (C=N), 1485, 1230, 1158. ^{1}H NMR (400 MHz, DMSO-*d*$_6$), δ, ppm (*J*, Hz): 1.17-1.29 (1H, m, C_6H_{11}); 1.30-1.46 (4H, m, C_6H_{11}); 1.35 (3H, s, 5-CH_3); 1.37 (3H, s, 5-CH_3); 1.66-1.84 (5H, m, C_6H_{11}); 2.19 (3H, s, 5-CH_3 ArOH); 2.86-3.00 (1H, m, Ar-C*H*-(CH_2)$_5$); 5.32 (1H, s, 2-H); 6.97 (1H, d, ^4J = 1.6, H-6′ ArOH); 7.04 (1H, d, ^4J = 1.6, H-2′ ArOH); 7.65 (2H, *AA′BB′*, *J* = 8.4, H_A: H-3′,5′ ArBr); 7.78 (2H, *AA′BB′*, *J* = 8.4, H_B: H-2′,6′ ArBr); 7.80 (1H, s, N-OH); 8.03 (1H, s, Ar-OH). ^{13}C NMR (100 MHz, DMSO-*d*$_6$), δ, ppm: 16.4 (5-CH_3); 17.0 (Ar-CH_3); 25.2 (5-CH_3); 25.9; 26.6; 32.9; 33.0 (4 CH_2); 36.5 (CH in C_6H_{11}); 70.4 (C-5); 89.6 (C-2); 123.8 (C-2′ ArOH); 123.8; 124.2 (C-Br and Me-*C* ArOH); 127.3 (C-6′ ArOH); 129.5 (C-2′,6′ ArBr); 131.6 (C-3′,5′ ArBr); 131.3; 132.0 (*C*(ArBr)-Het and *C*(ArOH)-Het); 133.4 (C-5′ ArOH); 151.6 (C-OH); 174.6 (C-4).

4-(4-Bromophenyl)-2-(4-hydroxy-3,5-diisopropylphenyl)-5,5-dimethyl-2,5-dihydro-1*H*-imidazol-1-ol (20m). Colorless powder, yield 2.08 g (78%), m.p. 195-197 °C (MeOH, dec.). Elemental analysis: found: C, 62.31; H, 6.43; Br, 17.94; N, 6.51; calcd. for $C_{23}H_{29}BrN_2O_2$: C, 62.02; H, 6.56; Br, 17.94; N, 6.29%. IR-spectrum, ν, cm⁻¹, (KBr): 3428, 3285 (OH), 2965, 2870 (CH), 1601 (C=N), 1467, 1289, 1203. ^{1}H NMR (400 MHz, DMSO-*d*$_6$), δ, ppm (*J*, Hz): 1.15 (12H, d, *J* = 6.8, C*H*$_3$-CH-C*H*$_3$); 1.35 (3H, s, 5-CH_3); 1.37 (3H, s, 5-CH_3); 3.30 (2H, septet, *J* = 6.8, CH_3-C*H*-CH_3); 5.35 (1H, s, H-2); 7.05 (2H, s, H-2,6 ArOH); 7.66

(2H, *AA′BB′*, *J* = 8.4, H_A: H-3′,5′ ArBr); 7.78 (2H, *AA′BB′*, *J* = 8.4, H_B: H-2′,6′ ArBr); 7.83 (1H, s, N-OH); 8.01 (1H, s, Ar-OH). ^{13}C NMR (100 MHz, DMSO-d_6), δ, ppm: 16.5 (5-CH_3); 23.1 (Ar-CH-*C*H_3); 25.3 (5-CH_3); 26.4 (Ar-*C*H-CH_3); 70.6 (C-5); 89.8 (C-2); 122.7 (C-2′,6' ArOH); 124.2 (C-Br); 129.5 (C-2′,6′ ArBr); 131.7 (C-3′,5′ ArBr); 131.7; 132.1 (*C*(Ar)-Het and *C*(Ph)-Het); 134.6 (*C*(Ar)-CH-CH_3); 150.4 (C-OH); 174.8 (C-4).

4-(4-Bromophenyl)-2-(3,5-dicyclohexyl-4-hydroxyphenyl)-5,5-dimethyl-2,5-dihydro-1*H*-imidazol-1-ol (20n). Colorless powder, yield 2.80 g (89%), m.p. 208-210 °C (EtOH, dec.). Elemental analysis: found: C, 66.17; H, 7.26; Br, 15.35; N, 5.23; calcd. for $C_{29}H_{37}BrN_2O_2$: C, 66.28; H, 7.10; Br, 15.20; N, 5.33%. IR-spectrum, ν, cm^{-1}, (KBr): 3269 (OH), 2926, 2850 (CH), 1591 (C=N), 1466, 1447, 1284, 1239. ^{1}H NMR (400 MHz, DMSO-d_6), δ, ppm (*J*, Hz): 1.16-1.30 (2H, m, C_6H_{11}); 1.30-1.46 (8H, m, C_6H_{11}); 1.35 (3H, s, 5-CH_3); 1.38 (3H, s, 5-CH_3); 1.67-1.83 (10H, m, C_6H_{11}); 2.88-2.98 (2H, m, Ar-C*H*-(CH_2)$_5$); 5.33 (1H, s, 2-H); 7.02 (2H, s, H-2,6 ArOH); 7.66 (2H, *AA′BB′*, *J* = 8.8, H_A: H-3′,5′ ArBr); 7.79 (2H, *AA′BB′*, *J* = 8.8, H_B: H-2′,6′ ArBr); 7.82 (1H, s, N-OH); 7.92 (1H, s, Ar-OH). ^{13}C NMR (100 MHz, DMSO-d_6), δ, ppm: 16.4 (5-CH_3); 25.3 (5-CH_3); 25.9; 26.6; 33.1; 33.2 (4 CH_2); 36.5 (CH in C_6H_{11}); 70.5 (C-5); 89.8 (C-2); 123.2 (C-2′,6′ ArOH); 124.2 (C-Br); 129.5 (C-2′,6′ ArBr); 131.6 (C-3′,5′ ArBr); 131.5; 132.0 (*C*(ArBr)-Het and *C*(ArOH)-Het); 133.8 (C-3′,5′ ArOH); 150.3 (C-OH); 174.6 (C-4).

4-(4-Bromophenyl)-2-(3,5-di-*tert*-butyl-4-hydroxyphenyl)-5,5-dimethyl-2,5-dihydro-1*H*-imidazol-1-ol (20o). Colorless fine crystals, yield 2.52 g (88%), m.p. 203-204 °C (MeOH, dec.). Elemental analysis: found: C, 63.50; H, 6.91; Br, 17.10; N, 5.68; calcd. for $C_{25}H_{33}BrN_2O_2$: C, 63.42; H, 7.03; Br, 16.88; N, 5.92%. IR-spectrum, ν, cm^{-1}, (KBr): 3627, 3244, (OH), 2958 (CH), 1612 (C=N), 1436, 1215. ^{1}H NMR (300 MHz, DMSO-d_6), δ, ppm (*J*, Hz): 1.38 (3H, s, 5-CH_3); 1.40 (21H, s, *t*-Bu and 5-CH_3); 5.36 (1H, s, H-2); 6.89 (1H, s, Ar-OH); 7.19 (2H, s, H-2′,6′ ArOH); 7.67 (2H, *AA′BB′*, *J* = 8.6, H_A: H-3′,5′ ArBr); 7.79 (2H, *AA′BB′*, *J* = 8.6, H_B: H-2′,6′ ArBr); 7.83 (1H, s, N-OH). ^{13}C NMR (75 MHz, DMSO-d_6), δ, ppm: 16.4 (5-CH_3); 25.3 (5-CH_3); 30.4 (C(CH_3)$_3$); 34.5 (*C*(CH_3)$_3$); 70.4 (C-5); 89.9 (C-2); 124.2 (C-2′,6′ ArOH); 124.2 (C-Br); 129.4 (C-2′,6′ ArBr); 131.7 (C-3′,5′ ArBr); 131.6; 132.1 (*C*(ArBr)-Het and *C*(ArOH)-Het); 138.5 (C-3′,5′ ArOH); 153.5 (C-OH); 174.8 (C-4).

2-(4-Hydroxy-3,5-dimethylphenyl)-4-(4-hydroxyphenyl)-5,5-dimethyl-2,5-dihydro-1*H*-imidazol-1-ol (20p). Colorless fine needles, yield 1.33 g (68%), m.p. 231.3-231.5 °C (EtOH). Elemental analysis: found: C 69.70; H 6.54; N 8.50; calcd. for $C_{19}H_{22}N_2O_3$: C, 69.92; H, 6.79; N, 8.58%. IR-spectrum, ν, cm^{-1}, (KBr): 3261 (OH), 2984, 2935 (CH), 2627, 2569 (OH), 1610, 1601, 1576 (C=N and C=C), 1518, 1286, 1213, 1155. ^{1}H NMR (400 MHz, DMSO-d_6), δ, ppm (*J*, Hz): 1.33 (3H, s, 5-CH_3); 1.39 (3H, s, 5-CH_3); 2.18 (6H, s, 3,5-CH_3 Ar); 5.23 (1H, s, H-2); 6.83 (2H, *AA′BB′*, *J* = 8.5, H_A: H-3′,5′ 4-ArOH); 6.98 (2H, s, H-2,6 2-ArOH); 7.68 (1H, s, N-OH); 7.72 (2H, *AA′BB′*, *J* = 8.5, H_B: H-2′,6′ 4-ArOH); 8.15 (1H, br.s, 2-Ar-OH); 9.99 (1H, br.s, 4-Ar-OH). ^{13}C NMR (100 MHz, DMSO-d_6), δ, ppm: 16.6 (5-CH_3); 16.8 (Ar-*C*H_3); 25.7 (5-CH_3); 70.1 (C-5); 88.9 (C-2); 115.3 (C-3′,5′ 4-ArOH); 123.5 (CH_3-*C* Ar); 123.8 (C-1′ 4-ArOH); 128.0; 129.3 (C-2′,6′ 2-ArOH and 4-ArOH); 131.8 (C-1′ 2-ArOH); 152.7 (C-OH 2-ArOH); 159.6 (C-OH 4-ArOH); 174.7 (C-4).

2-(4-Hydroxy-3,5-diisopropylphenyl)-4-(4-hydroxyphenyl)-5,5-dimethyl-2,5-dihydro-1*H*-imidazol-1-ol (20q). Faint yellow powder, yield 1.61 g (70%), m.p. 218.7 °C (H_2O/$CHCl_3$, dec.). Elemental analysis: found: C, 70.64; H, 7.76; N, 7.13; calcd. for $C_{23}H_{30}N_2O_3 \times ½H_2O$: C, 70.56; H, 7.98; N, 7.16%. IR-spectrum, ν, cm^{-1}, (KBr): 3577, 3431, 3286, 3078, (OH), 2964, 2931, 2870 (CH), 1608 (C=N), 1560, 1518, 1464, 1298, 1161. ^{1}H NMR (400 MHz, DMSO-d_6), δ, ppm (*J*, Hz): 1.15 (12H, d, *J* = 5.6, C*H*$_3$-CH-C*H*$_3$); 1.33 (3H, s, 5-CH_3); 1.38 (3H, s, 5-CH_3); 3.30 (2H, septet, *J* = 5.6, CH_3-C*H*-CH_3); 5.28 (1H, s, H-2); 6.83 (2H, *AA′BB′*, *J* = 7.1, H_A: H-3′,5′ 4-ArOH); 7.05 (2H, s, H-2,6 2-ArOH); 7.70 (2H, *AA′BB′*, *J* = 7.1, H_B: H-2′,6′ 4-ArOH); 7.72 (1H, s, N-OH); 7.95 (1H, br.s, 2-Ar-OH); 9.97 (1H, br.s, 4-Ar-OH). ^{13}C NMR (100 MHz, DMSO-d_6), δ, ppm: 16.6 (5-CH_3); 23.2 (Ar-CH-CH_3); 25.7 (5-CH_3); 26.4 (Ar-CH-CH_3); 70.3 (C-5); 89.4 (C-2); 115.4 (C-3′,5′ 4-ArOH); 122.8 (C-2′,6' 2-ArOH); 123.9 (C-1′ 4-ArOH); 129.3 (C-2′,6′ 4-

ArOH); 132.3 (C-1 2-ArOH); 134.6 (C-3,5 2-ArOH); 150.3 (C-OH 2-ArOH); 159.6 (C-OH 4-ArOH); 174.8 (C-4).

2-(3,5-Dicyclohexyl-4-hydroxyphenyl)-4-(4-hydroxyphenyl)-5,5-dimethyl-2,5-dihydro-1*H*-imidazol-1-ol (20r). Colorless powder, yield 2.03 g (73%), m.p. 214.5-215.0 °C (EtOH). Elemental analysis: found: C, 75.13; H, 8.13; N, 5.89; calcd. for $C_{29}H_{38}N_2O_3$: C, 75.29; H, 8.28; N, 6.06%. IR-spectrum, ν, cm^{-1}, (KBr): 3279 (OH), 2924, 2850 (CH), 1603 (C=N), 1448, 1284, 1234. ^{1}H NMR (300 MHz, DMSO-d_6), δ, ppm (*J*, Hz): 1.10-1.48 (10H, m, C_6H_{11}); 1.33 (3H, s, 5-CH_3); 1.39 (3H, s, 5-CH_3); 1.63-1.86 (10H, m, C_6H_{11}); 2.80-3.05 (2H, m, Ar-C*H*-$(CH_2)_5$); 5.26 (1H, s, 2-H); 6.84 (2H, *AA′BB′*, *J* = 8.3, H_A: H-3′,5′ 4-ArOH); 7.01 (2H, s, H-2,6 2-ArOH); 7.71 (1H, s, N-OH); 7.72 (2H, *AA′BB′*, *J* = 8.3, H_B: H-2′,6′ 4-ArOH); 7.90 (1H, br.s, 2-Ar-OH); 9.97 (1H, br.s, 4-Ar-OH). ^{13}C NMR (75 MHz, DMSO-d_6), δ, ppm: 16.6 (5-CH_3); 25.7 (5-CH_3); 26.0; 26.7; 33.2; 33.3 (4 CH_2); 36.6 (CH in C_6H_{11}); 70.3 (C-5); 89.4 (C-2); 115.4 (C-3′,5′ 4-ArOH); 123.3 (C-2′,6′ 2-ArOH); 123.9 (C-1′ 4-ArOH); 129.3 (C-2′,6′ 4-ArOH); 132.2 (C-1′ 2-ArOH); 133.8 (C-3′,5′ 2-ArOH); 150.2 (C-OH 2-ArOH); 159.6 (C-OH 4-ArOH); 174.7 (C-4).

2-(3,5-Di-*tert*-butyl-4-hydroxyphenyl)-4-(4-hydroxyphenyl)-5,5-dimethyl-2,5-dihydro-1*H*-imidazol-1-ol methanolate (20s×CH_3OH). Colorless plates, yield 2.02 g (76%), m.p. 237.9 °C (MeOH, dec.). Elemental analysis: found: C, 70.83; H, 8.49; N, 6.54; calcd. for $C_{25}H_{34}N_2O_3 \times CH_3OH$: C, 70.56; H, 8.65; N, 6.33%. IR-spectrum, ν, cm^{-1}, (KBr): 3611 (OH), 2957 (CH), 1609, 1590 (C=N), 1536, 1513, 1462, 1422, 1362, 1194. ^{1}H NMR (500 MHz, DMSO-d_6), δ, ppm (*J*, Hz): 1.35 (3H, s, 5-CH_3); 1.39 (3H, s, 5-CH_3); 1.40 (18H, s, *t*-Bu); 3.18 (3H, s, C*H*$_3$OH); 4.07 (1H, br.s, CH_3O*H*); 5.28 (1H, s, H-2); 6.79 (1H, s, 2-Ar-O*H*); 6.83 (2H, *AA′BB′*, J_{AB} = 8.6, H-3,5 4-Ar-OH); 7.19 (2H, s, H-2,6 2-ArOH); 7.68 (1H, s, N-OH); 7.71 (2H, *AA′BB′*, J_{AB} = 8.6, H-2,6 4-Ar-OH); 9.89 (1H, br.s, 4-Ar-O*H*). ^{13}C NMR (125 MHz, DMSO-d_6), δ, ppm: 16.8 (5-CH_3); 26.0 (5-CH_3); 30.7 ($C(CH_3)_3$); 34.7 (*C*$(CH_3)_3$); 48.9 (CH_3OH); 70.5 (C-5); 89.8 (C-2); 115.5 (C-3′,5′ 4-ArOH); 124.1 (C-1′ 4-ArOH); 124.4 (C-2′,6′ 2-ArOH); 129.4 (C-2′,6′ 4-ArOH); 132.4 (C-1′ 2-ArOH); 138.6 (C-3′,5′ 2-ArOH); 153.6 (C-OH 2-ArOH); 159.8 (C-OH 4-ArOH); 175.0 (C-4).

2-(4-Hydroxy-3,5-dimethylphenyl)-4,4-dimethyl-5-phenyl-4*H*-imidazole 3-oxide (21a). Yellow needles, yield 1.34 g (87%), m.p. 208-210 °C (EtOH, dec.). Elemental analysis: found: C, 74.05; H, 6.53; N, 9.12; calcd. for $C_{19}H_{20}N_2O_2$: C, 74.00; H, 6.54; N, 9.08%. IR-spectrum, ν, cm^{-1}, (KBr): 3552, 3393 (OH), 2940 (CH), 1603 (C=N), 1550, 1522, 1327, 1179. UV (EtOH), λ_{max}nm, (lg ε): 301 (4.37), 385 (3.63). ^{1}H NMR (300 MHz, $CDCl_3$), δ, ppm (*J*, Hz): 1.75 (6H, s, 4,4-CH_3); 2.31 (6H, s, 3,5-CH_3 Ar); 5.33 (1H, br.s, Ar-OH); 7.46-7.55 (3H, m, Ph); 8.06-8.12 (2H, m, Ph); 8.47 (2H, s, H-2′,6′ Ar).

2-(3-Cyclohexyl-4-hydroxy-5-methylphenyl)-4,4-dimethyl-5-phenyl-4*H*-imidazole 3-oxide (21b). Bright yellow needles, yield 1.53 g (81%), m.p. 211-212 °C (MeCN, dec.). Elemental analysis: found: C, 76.28; H, 7.43; N, 7.34; calcd. for $C_{24}H_{28}N_2O_2$: C, 76.56; H, 7.50; N, 7.44%. IR-spectrum, ν, cm^{-1}, (KBr): 3406 (OH), 3060 (CH), 2931, 2850, 1604 (C=N), 1545, 1472, 1326, 1187. UV (EtOH), λ_{max}nm, (lg ε): 301 (4.33), 386 (3.60). ^{1}H NMR (400 MHz, DMSO-d_6), δ, ppm (*J*, Hz): 1.20-1.48 (5H, m, CH(C*H*$_2$)$_5$; 1.64 (6H, s, 4,4-CH_3); 1.69-1.87 (5H, m, CH(C*H*$_2$)$_5$); 2.27 (3H, s, 5-CH_3 Ar); 2.93-3.03 (1H, m, C*H*$(CH_2)_5$); 7.54-7.62 (3H, m, Ph); 8.14-8.19 (2H, m, Ph); 8.30 (1H, d, ^4J = 2.0, H-6′ ArOH); 8.50 (1H, d, ^4J = 2.0, H-2′ ArOH); 8.83 (1H, br.s, OH). ^{13}C NMR (100 MHz, DMSO-d_6), δ, ppm: 17.1 (Ar-CH_3); 23.6 (4-CH_3); 25.8; 26.5; 32.9 (3 CH_2); 36.4 (CH in C_6H_{11}); 80.3 (C-4); 118.9 (C-1′ Ar); 123.6 (C-2′ Ar); 124.3 (C-3′ Ar); 127.2; 129.2; 131.7 (3CH Ph); 127.3 (C-6′ Ar); 130.2; 133.9 (C-5′ Ar and C-1 Ph); 144.3 (C-2); 154.3 (C-OH); 174.9 (C-5).

2-(4-Hydroxy-3,5-diisopropylphenyl)-4,4-dimethyl-5-phenyl-4*H*-imidazole 3-oxide (21c). Bright yellow tiny needles, yield 1.55 g (87%), m.p. 212-215 °C (EtOH, dec.). Elemental analysis: found: C, 75.61; H, 7.75; N, 7.77; calcd. for $C_{23}H_{28}N_2O_2$: C, 75.79; H, 7.74; N, 7.69%. IR-spectrum, ν, cm^{-1}, (KBr): 3197 (OH), 2960, 2871 (CH) 1600 (C=N), 1541, 1461, 1430, 1186, 1124. ^{1}H NMR (300 MHz, $CDCl_3$), δ, ppm (*J*, Hz): 1.30 (12H, d, *J* = 6.6, (C*H*$_3$-CH-C*H*$_3$)$_2$); 1.76 (6H, s, 4,4-CH_3); 3.25 (2H, septet, *J* = 6.6, (CH_3-C*H*-CH_3)$_2$); 6.12 (1H, s, OH); 7.40-7.51 (3H, m, Ph); 8.02-8.10 (2H, m, Ph); 8.59 (2H, s, H-2′,6′ ArOH).

^{13}C NMR (75 MHz, $CDCl_3$), δ, ppm: 25.0 (Ar-CH-*C*H_3); 26.5 (4-CH_3); 29.6 (Ar-CH-CH_3); 83.0 (C-4); 122.2 (C-1′ Ar), 126.6 (C-2′,6′ Ar); 129.7; 131.3; 134.0 (3CH Ph); 133.0 (C-1 Ph); 136.3 (C-3′,5′ Ar); 149.1 (C-2); 155.2 (C-OH); 178.7 (C-5).

2-(3,5-Dicyclohexyl-4-hydroxyphenyl)-4,4-dimethyl-5-phenyl-4*H*-imidazole 3-oxide (21d). Yellow powder, yield 2.07 g (93%), m.p. 249-250 °C (MeOH, dec.). Elemental analysis: found: C, 78.49; H, 8.15; N, 6.31; calcd. for $C_{29}H_{36}N_2O_2$: C, 78.34; H, 8.16; N, 6.30%. IR-spectrum, ν, cm^{-1}, (KBr): 3156 (OH), 2922, 2850 (CH), 1596 (C=N), 1536, 1449, 1431, 1195. UV (EtOH), λ_{max}nm, (lg ε): 303 (4.46), 387 (3.69). ^{1}H NMR (300 MHz, $CDCl_3$), δ, ppm (*J*, Hz): 1.23-1.67 (14H, m, CH(C*H_2*)$_5$); 1.75 (6H, s, 4,4-CH_3); 1.74-1.96 (6H, m, CH(C*H_2*)$_5$); 2.72-2.83 (2H, m, C*H*(CH_2)$_5$); 5.22 (1H, s, OH); 7.48-7.55 (3H, m, Ph); 8.07-8.13 (2H, m, Ph); 8.57 (2H, s, H-2′,6′ ArOH).

2-(3,5-Di-*tert*-butyl-4-hydroxyphenyl)-4,4-dimethyl-5-phenyl-4*H*-imidazole 3-oxide (21e). Bright yellow needles, yield 1.89 g (97%), m.p. 240-242 °C (EtOH, dec.). Elemental analysis: found: C, 76.59; H, 8.22; N, 7.26; calcd. for $C_{25}H_{32}N_2O_2$: C, 76.49; H, 8.22; N, 7.14%. IR-spectrum, ν, cm^{-1}, (KBr): 3602 (OH), 2955 (CH), 1598 (C=N), 1531, 1423, 1374, 1241. ^{1}H NMR (300 MHz, $CDCl_3$), δ, ppm (*J*, Hz): 1.51 (18H, s, *t*-Bu); 1.75 (6H, s, 4,4-CH_3); 5.63 (1H, s, OH); 7.45-7.53 (3H, m, Ph); 8.06-8.11 (2H, m, Ph); 8.75 (2H, s, H-2′,6′ Ar). ^{13}C NMR (75 MHz, $CDCl_3$), δ, ppm: 23.9 (4-CH_3); 29.8 (C(CH_3)$_3$); 34.1 (*C*(CH_3)$_3$); 80.2 (C-4); 118.8 (C-1′ Ar); 125.2 (C-2′,6′ Ar); 126.9; 128.6; (2CH Ph); 130.4 (C-1 Ph); 131.2 (CH Ph); 135.5 (C-3′,5′ Ar); 146.1 (C-2); 155.7 (C-OH); 175.4 (C-5).

5-(4-Fluorophenyl)-2-(4-hydroxy-3,5-dimethylphenyl)-4,4-dimethyl-4*H*-imidazole 3-oxide (21f). Yellowish green needles, yield 1.40 g (86%), m.p. 205-210 °C (*t*-BuOMe, dec.). Elemental analysis: found: C, 69.89; H, 5.87; F, 5.75; N, 8.74; calcd. for $C_{19}H_{19}FN_2O_2$: C, 69.92; H, 5.87; F, 5.82; N, 8.58%. IR-spectrum, ν, cm^{-1}, (KBr): 3075 (OH), 2946 (CH), 1603 (C=N), 1550, 1507, 1149. UV (EtOH), λ_{max}nm, (lg ε): 301 (4.48), 383 (3.73). ^{1}H NMR (400 MHz, DMSO-*d*$_6$), δ, ppm (*J*, Hz): 1.62 (6H, s, 4,4-CH_3); 2.25 (6H, s, 3,5-CH_3 2-ArOH); 7.38 (2H, ddd, $^3J_{HF}$ = 8.5, ^3J = 8.2, ^4J = 1.5, H-3″,5″ ArF); 8.22 (2H, ddd, ^3J = 8.2, $^4J_{HF}$ = 5.6, ^4J = 1.5, H-2″,6″ ArF); 8.34 (2H, s, H-2′,6′ 2-ArOH); 8.95 (1H, br.s, OH).

2-(3-Cyclohexyl-4-hydroxy-5-methylphenyl)-5-(4-fluorophenyl)-4,4-dimethyl-4*H*-imidazole 3-oxide (21g). Yellowish green needles, yield 1.69 g (86%), m.p. 236-238 °C (MeOH, dec.). Elemental analysis: found: C, 73.03; H, 6.85; F, 4.92; N, 7.27; calcd. for $C_{24}H_{27}FN_2O_2$: C, 73.03; H, 6.90; F, 4.82; N, 7.10%. IR-spectrum, ν, cm^{-1}, (KBr): 3408 (OH), 2931, 1602 (C=N), 1541, 1504, 1186, 1150. UV (EtOH), λ_{max}nm, (lg ε): 302 (4.48), 388 (3.74). ^{1}H NMR (400 MHz, $CDCl_3$), δ, ppm (*J*, Hz): 1.22-1.60 (5H, m, CH(C*H_2*)$_5$); 1.73 (6H, s, 4,4-CH_3), 1.74-1.93 (5H, m, CH(C*H_2*)$_5$); 2.32 (3H, s, 5-CH_3 2-ArOH), 2.76-2.86 (1H, m, C*H*(CH_2)$_5$); 5.40 (1H, s, OH); 7.19 (2H, ddd, $^3J_{HF}$ = 8.5, ^3J = 8.2, ^4J = 1.5, H-3″,5″ ArF); 8.10 (2H, ddd, ^3J = 8.2, $^4J_{HF}$ = 5.6, ^4J = 1.5, H-2″,6″ ArF); 8.44 (1H, d, ^4J = 1.6, H-6′ ArOH); 8.55 (1H, d, ^4J = 1.6, H-2′ ArOH).

5-(4-Fluorophenyl)-2-(4-hydroxy-3,5-diisopropylphenyl)-4,4-dimethyl-4*H*-imidazole 3-oxide (21h). Yellowish green crystals, yield 1.87 g (96%), m.p. 220-231 °C (MeCN, dec.). Elemental analysis: found: C, 72.21; H, 7.10; F, 4.95; N, 7.30; calcd. for $C_{23}H_{27}FN_2O_2$: C, 72.23; H, 7.12; F, 4.97; N 7.32%. IR-spectrum, ν, cm^{-1}, (KBr): 3227 (OH), 2960 (CH), 1603 (C=N), 1549, 1509, 1463, 1432, 1194, 1144. ^{1}H NMR (300 MHz, DMSO-*d*$_6$), δ, ppm (*J*, Hz): 1.22 (12H, d, *J* = 6.9, C*H_3*-CH-C*H_3*); 1.65 (6H, s, 4-CH_3); 3.37 (2H, septet, *J* = 6.9, CH_3-C*H*-CH_3); 7.39 (2H, ddd, $^3J_{HF}$ = 8.0, ^3J = 7.5, ^4J = 1.5, H-3′,5′ 5-ArF); 8.24 (2H, ddd, ^3J = 7.5, $^4J_{HF}$ = 5.0, ^4J = 1.5, H-2′,6′ ArF); 8.48 (2H, s, 2,6-H 2-ArOH); 8.81 (1H, s, OH). ^{13}C NMR (75 MHz, DMSO-*d*$_6$), δ, ppm: 23.2 (Ar-CH_3); 23.8 (4,4-CH_3); 26.6 (CH_3-CH-CH_3); 80.6 (C-4); 116.6 (d, $^2J_{CF}$ = 22, C-3′,5′ ArF); 119.5 (C-1 ArOH); 123.1 (C-2,6 ArOH); 127.3 (d, $^4J_{CF}$ = 3.2, C-1′ ArF); 130.2 (d, $^3J_{CF}$ = 8.8, C-2′,6′ ArF); 135.2 (C-3,5 ArOH); 144.8 (C-2); 153.4 (C-OH); 164.3 (d, $^1J_{CF}$ = 250, C-4′ ArF); 174.1 (C-5).

2-(3,5-Dicyclohexyl-4-hydroxyphenyl)-5-(4-fluorophenyl)-4,4-dimethyl-4*H*-imidazole 3-oxide (21i). Yellowish green crystals, yield 2.22 g (96%), m.p. 255-257 °C (MeOH, dec.). Elemental analysis: found: C, 75.39; H, 7.61; F, 4.32; N, 6.08; calcd. for $C_{29}H_{35}FN_2O_2$: C, 75.29; H, 7.63; F, 4.11; N, 6.06%. IR-

spectrum, ν, cm⁻¹, (KBr): 3109, 2924 (CH), 1604 (C=N), 1536, 1508, 1431, 1238, 1193. ^{1}H NMR (300 MHz, DMSO-d_6), δ, ppm (*J*, Hz): 1.20-1.51 (10H, m, CH(C*H*$_2$)$_5$); 1.63 (6H, s, 4,4-CH$_3$); 1.68-1.86 (10H, m, CH(C*H*$_2$)$_5$); 2.93-3.05 (2H, m, C*H*(CH$_2$)$_5$); 7.37 (2H, ddd, $^3J_{HF}$ = 8.0, ^3J = 7.3, ^4J = 1.6, H-3′,5′ 5-ArF); 8.22 (2H, ddd, ^3J = 7.5, $^4J_{HF}$ = 5.0, ^4J = 1.4, H-2′,6′ ArF); 8.41 (2H, s, 2,6-H 2-ArOH); 8.68 (1H, s, OH).

2-(3,5-Di-*tert*-butyl-4-hydroxyphenyl)-5-(4-fluorophenyl)-4,4-dimethyl-4*H*-imidazole 3-oxide methanolate (21j×CH$_3$OH). Yellowish green crystals, yield 1.99 g (90%), m.p. 230-232 °C (MeOH, dec.). Elemental analysis: found: C, 71.01; H, 7.96; N, 6.43; calcd. for $C_{25}H_{31}FN_2O_2$×CH_3OH: C, 70.56; H, 7.97; N, 6.33%. IR-spectrum, ν, cm⁻¹, (KBr): 3434 (OH), 2963 (CH), 1603, 1540 (C=N), 1508, 1421, 1369, 1240. ^{1}H NMR (300 MHz, CDCl$_3$), δ, ppm (*J*, Hz): 1.54 (18H, s, C(CH$_3$)$_3$); 1.77 (6H, s, 4,4-CH$_3$); 3.49 (3H, s, C*H*$_3$OH); 5.68 (1H, s, OH); 7.24 (2H, ddd, $^3J_{HF}$ = 8.0, ^3J = 7.5, ^4J = 1.5, H-3′,5′ 5-ArF); 8.12 (2H, ddd, ^3J = 7.5, $^4J_{HF}$ = 5.0, ^4J = 1.4, H-2′,6′ ArF); 8.77 (2H, s, 2,6-H 2-ArOH). ^{13}C NMR (75 MHz, CDCl$_3$), δ, ppm: 23.8 (4,4-CH$_3$); 29.8 (C(CH$_3$)$_3$); 33.9 (*C*(CH$_3$)$_3$); 50.3 (CH$_3$OH); 80.1 (C-4); 115.8 (d, $^2J_{CF}$ = 22, C-3′,5′ ArF); 118.7 (C-1 ArOH); 125.2 (C-2,6 ArOH); 126.8 (d, $^4J_{CF}$ = 3.4, C-1′ ArF); 129.1 (d, $^3J_{CF}$ = 8.6, C-2′,6′ ArF); 135.5 (C-3,5 ArOH); 146.1 (C-2); 155.8 (C-OH); 164.3 (d, $^1J_{CF}$ = 253, C-4′ ArF); 175.0 (C-5).

5-(4-Bromophenyl)-2-(4-hydroxy-3,5-dimethylphenyl)-4,4-dimethyl-4*H*-imidazole 3-oxide (21k). Bright yellow crystals, yield 1.77 g (91%), m.p. 228-230 °C (AcOH, dec.). Elemental analysis: found: C, 58.99; H, 4.88; Br, 20.31; N, 7.34; calcd. for $C_{19}H_{19}BrN_2O_2$: C, 58.93; H, 4.95; Br, 20.63; N, 7.23%. IR-spectrum, ν, cm⁻¹, (KBr): 3421 (OH), 2943 (CH), 1589 (C=N), 1539, 1476, 1178. UV (EtOH), λ_{max}nm, (lg ε): 306 (4.51), 396 (3.76). ^{1}H NMR (400 MHz, DMSO-d_6), δ, ppm (*J*, Hz): 1.61 (6H, s, C(CH$_3$)$_2$); 2.24 (6H, s, 3,5-CH$_3$ Ar); 7.73 (2H, *AA′BB′*, *J* = 8.8, H$_A$: H-3′,5′ 5-ArBr); 8.07 (2H, *AB*, *J* = 8.8, H$_B$: H-2′,6′ 5-ArBr); 8.32 (2H, s, H-2,6 2-Ar); 9.05 (1H, br.s, OH). ^{13}C NMR (100 MHz, DMSO-d_6), δ, ppm: 16.8 (Ar-CH$_3$); 23.5 (4,4-CH$_3$); 80.4 (C-4); 118.5 (C-1 ArOH); 124.2 (Ar-*C*-Me); 125.5 (C-Br); 127.7, 129.2 (CH Ar); 129.3 (C-1′ ArBr); 132.3 (CH Ar); 144.4 (C-2); 155.6 (C-OH); 174.0 (C-5).

5-(4-Bromophenyl)-2-(3-cyclohexyl-4-hydroxy-5-methylphenyl)-4,4-dimethyl-4*H*-imidazole 3-oxide (21l). Dark yellow needles, yield 2.15 g (94%), m.p. 238-239 °C (MeOH, dec.). Elemental analysis found:C, 63.21; H, 6.05; Br, 17.54; N, 6.12; calcd. for $C_{24}H_{27}BrN_2O_2$: C, 63.30; H, 5.98; Br, 17.55; N, 6.15%. IR-spectrum, ν, cm⁻¹, (KBr): 3370 (OH), 2930 (CH), 1602 (C=N), 1587, 1543, 1468, 1181. ^{1}H NMR (300 MHz, CDCl$_3$), δ, ppm (*J*, Hz): 1.20-1.65 (5H, m, CH(C*H*$_2$)$_5$); 1.72 (6H, s, 4,4-CH$_3$); 1.73-1.95 (5H, m, CH(C*H*$_2$)$_5$); 2.33 (3H, s, 5-CH$_3$ 2-ArOH); 2.73-2.88 (1H, m, C*H*(CH$_2$)$_5$); 5.25 (1H, s, OH); 7.63 (2H, *AA′BB′*, *J* = 8.7, H$_A$: H-3′,5′ 5-ArBr); 7.95 (2H, *AB*, *J* = 8.7, H$_B$: H-2′,6′ 5-ArBr); 8.43 (1H, d, ^4J = 1.5, H-6′ ArOH); 8.56 (1H, d, ^4J = 1.5, H-2′ ArOH).

5-(4-Bromophenyl)-2-(4-hydroxy-3,5-diisopropylphenyl)-4,4-dimethyl-4*H*-imidazole 3-oxide (21m). Dark yellow needles, yield 2.14 g (97%), m.p. 228-230 °C (70% aq. AcOH, dec.). Elemental analysis: found: C, 61.95; H, 6.16; Br, 18.03; N, 6.12; calcd. for $C_{23}H_{27}BrN_2O_2$: C, 62.31; H, 6.14; Br, 18.02; N, 6.32%. IR-spectrum, ν, cm⁻¹, (KBr): 3243 (OH), 2958 (CH), 1604, 1588 (C=N), 1546, 1460, 1432, 1194, 1138. ^{1}H NMR (300 MHz, CDCl$_3$), δ, ppm (*J*, Hz): 1.30 (12H, d, *J* = 6.9, C*H*$_3$-CH-C*H*$_3$); 1.73 (6H, s, 4-CH$_3$); 3.22 (2H, septet, *J* = 6.9, CH$_3$-C*H*-CH$_3$); 5.94 (1H, s, OH); 7.63 (2H, *AA′BB′*, *J* = 8.9, H$_A$: H-3′,5′ 5-ArBr); 7.95 (2H, *AA′BB′*, *J* = 8.9, H$_B$: H-2′,6′ 5-ArBr); 8.55 (2H, s, H-2,6 2-ArOH). ^{13}C NMR (75 MHz, CDCl$_3$), δ, ppm: 22.2 (Ar-CH-CH$_3$); 23.7 (4,4-CH$_3$); 26.9 (Ar-CH-CH$_3$); 80.2 (C-4); 119.4 (C-1 ArOH); 123.8 (C-2,6 ArOH); 126.0 (C-Br); 128.3 (C-2′,6′ ArBr); 129.2 (C-1′ ArBr); 131.9 (C-3′,5′ ArBr); 133.6 (C-3,5 ArOH); 146.3 (C-2); 152.5 (C-OH); 174.7 (C-5).

5-(4-Bromophenyl)-2-(3,5-dicyclohexyl-4-hydroxyphenyl)-4,4-dimethyl-4*H*-imidazole 3-oxide (21n). Dark yellow crystals, yield 2.62 g (100%), m.p. 248-250 °C (EtOH, dec.). Elemental analysis: found:C, 66.60; H, 6.81; Br, 15.30; N, 5.29; calcd. for $C_{29}H_{35}BrN_2O_2$: C, 66.53; H, 6.74; Br, 15.26; N, 5.35%. IR-spectrum, ν, cm⁻¹, (KBr): 3236 (OH), 2922 (CH), 1587 (C=N), 1539, 1432, 1195. ^{1}H NMR (400 MHz, CDCl$_3$), δ, ppm (*J*, Hz): 1.20-1.62 (10H, m, CH(C*H*$_2$)$_5$); 1.71 (6H, s, 4-CH$_3$); 1.75-1.95 (10H, m, CH(C*H*$_2$)$_5$); 2.72-2.86 (2H, m, C*H*(CH$_2$)$_5$); 5.66 (1H, s, OH); 7.63 (2H, *AA′BB′*, *J* = 7.2, H$_A$: H-3′,5′ 5-ArBr);

7.95 (2H, *AA′BB′*, *J* = 7.2, H_B: H-2′,6′ 5-ArBr); 8.53 (2H, s, 2,6-H 2-ArOH). ^{13}C NMR (100 MHz, $CDCl_3$), δ, ppm: 24.0 (4,4-CH_3); 26.0, 26.9, 32.9 (CH_2 in C_6H_{11}); 37.6 (*C*H in C_6H_{11}); 80.4 (C-4); 119.8 (C-1 Ar-OH); 124.5 (C-2,6 ArOH); 126.2 (C-Br); 128.6 (C-2′,6′ ArBr); 129.5 (C-1′ ArBr); 132.2 (C-3′,5′ ArBr); 133.0 (C-3,5 ArOH); 146.5 (C-2); 152.5 (C-OH); 174.8 (C-5).

5-(4-Bromophenyl)-2-(3,5-di-*tert*-butyl-4-hydroxyphenyl)-4,4-dimethyl-4*H*-imidazole 3-oxide acetate (21o×CH_3COOH). Dark yellow crystals, yield 2.19 g (93%), m.p. 230-232 °C (70% aq AcOH, dec.). Elemental analysis: found: C, 61.00; H, 6.60; N, 5.24; calcd. for $C_{25}H_{31}BrN_2O_2$×CH_3COOH: C, 61.02; H, 6.64; N, 5.27%. IR-spectrum, ν, cm^{-1}, (KBr): 3619 (OH), 2957 (CH), 1586 (C=N), 1530, 1422, 1237. ^{1}H NMR (300 MHz, $CDCl_3$), δ, ppm (*J*, Hz): 1.54 (18H, s, C(CH_3)$_3$); 1.77 (6H, s, 4-CH_3); 2.08 (3H, s, C*H*$_3$COOH); 5.72 (1H, br.s, OH); 7.65 (2H, *AA′BB′*, *J* = 8.7, H_A: H-3′,5′ 5-ArBr); 7.98 (2H, *AA′BB′*, *J* = 8.7, H_B: H-2′,6′ 5-ArBr); 8.75 (2H, s, 2,6-H 2-ArOH); 10.75 (1H, br.s, CH_3COO*H*). ^{13}C NMR (75 MHz, $CDCl_3$), δ, ppm: 20.7 (*C*H$_3$COOH); 23.9 (4,4-CH_3); 30.1 (C(CH_3)$_3$); 34.4(*C*(CH_3)$_3$); 80.5 (C-4); 118.7 (C-1 Ar-OH); 125.8 (C-2,6 ArOH); 126.4 (C-Br); 128.6 (C-2′,6′ ArBr); 129.4 (C-1′ ArBr); 132.2 (C-3′,5′ ArBr); 135.8 (C-3,5 ArOH); 147.2 (C-2); 156.3 (C-OH); 175.5 (C-5); 175.8 (CH_3COOH).

2-(4-Hydroxy-3,5-dimethylphenyl)-5-(4-hydroxyphenyl)-4,4-dimethyl-4*H*-imidazole 3-oxide (21p). Dark yellow crystals, yield 1.35 g (83%), m.p. 270.4 °C (chromatography, dec.). Elemental analysis: found: C, 70.52; H, 6.35; N, 8.49; calcd. for $C_{19}H_{20}N_2O_3$: C 70.35; H 6.21; N 8.64%. IR-spectrum, ν, cm^{-1}, (KBr): 3072, 2980, 2920, 2796, 2675, 2586, 2469 (CH, OH), 1605, 1556 (C=N, C=C), 1500, 1327, 1146. UV (EtOH), λ_{max}nm, (lg ε): 248 (3.99), 308 (4.40), 330 (4.33), 384 (3.91). ^{1}H NMR (500 MHz, $CDCl_3$+DMSO-*d*$_6$), δ, ppm (*J*, Hz): 1.62 (6H, s, 4-CH_3); 2.24 (6H, s, 3,5-CH_3 2-Ar); 3.38 (2H, br.s, 2(5)-Ar-OH); 6.88 (2H, *AA′BB′*, *J* = 8.6, H_A: H-3′,5′ 5-ArOH); 7.94 (2H, *AA′BB′*, *J* = 8.6, H_B: H-2′,6′ 5-ArOH); 8.33 (2H, s, H-2,6 2-ArOH). ^{13}C NMR (125 MHz, $CDCl_3$+DMSO-*d*$_6$), δ, ppm: 16.5 (Ar-CH_3); 23.8 (4,4-CH_3); 79.4 (C-4); 115.7 (C-3′,5′ 5-ArOH); 118.5 (C-1 2-ArOH); 121.5 (C-1′ 5-ArOH); 123.7 (CH_3-*C* 2-ArOH); 127.9; 129.0 (C-2′,6′ 2-ArOH and 4-ArOH); 144.8 (C-2); 155.4 (C-OH 2-ArOH); 160.8 (C-OH 4-ArOH); 175.4 (C-5).

2-(4-Hydroxy-3,5-diisopropylphenyl)-5-(4-hydroxyphenyl)-4,4-dimethyl-4*H*-imidazole 3-oxide (21q). Yellow green powder, yield 343 mg (90%), m.p. 299.7-299.9 °C ($CHCl_3$). Elemental analysis: found: C, 67.82; H 6.86; N 6.91; calcd. for $C_{23}H_{28}N_2O_3$×¼ $CHCl_3$: C 68.06; H 6.94; N 6.83%. IR-spectrum, ν, cm^{-1}, (KBr): 3495, 3063 (OH), 2962, 2871 (CH), 2820, 2767, 2690, 2631, 2515, 1605 (C=N), 1591 (C=C), 1552, 1512, 1468, 1433, 1284, 1188, 1144. UV (EtOH), λ_{max}nm, (lg ε): 249 (3.70), 308 (4.09), 331 (4.04), 386 (3.60). ^{1}H NMR (500 MHz, DMSO-*d*$_6$), δ, ppm (*J*, Hz): 1.22 (12H, d, *J* = 6.9, C*H*$_3$-CH-C*H*$_3$); 1.62 (6H, s, 4-CH_3); 3.41 (2H, septet, *J* = 6.9, CH_3-C*H*-CH_3); 6.95 (2H, br.s, H-3′,5′ 5-Ar-OH); 8.04 (2H, br.s, H-2′,6′ 5-Ar-OH); 8.48 (2H, s, H-2,6 2-ArOH); 8.77 (1H, br.s, 2-Ar-OH); 10.34 (1H, br.s, 5-Ar-OH). ^{13}C NMR (125 MHz, DMSO-*d*$_6$), δ, ppm: 22.9 (*C*H$_3$-CH-*C*H$_3$); 23.9 (4,4-CH_3); 26.3 (CH_3-CH-CH_3); 79.8 (C-4); 116.0 (C-3′,5′ 5-ArOH); 119.5 (C-1 2-ArOH); 121.7 (C-1′ 5-ArOH); 122.9 (C-2,6 2-ArOH); 129.5 (C-2′,6′ 5-ArOH); 134.9 (C-3,5 2-ArOH); 144.6 (C-2); 153.0 (C-OH 2-ArOH); 160.9 (C-OH 5-ArOH); 175.2 (C-5).

2-(3,5-Dicyclohexyl-4-hydroxyphenyl)-5-(4-hydroxyphenyl)-4,4-dimethyl-4*H*-imidazole 3-oxide methanolate (21r). Yellow green tiny needles, yield 2.09 g (85%), m.p. 278.8-279.2 °C (MeOH). Elemental analysis: found: C 73.00; H 8.09; N 5.56; calcd. for $C_{29}H_{36}N_2O_3$×CH_3OH: C, 73.14; H, 8.18; N, 5.69%. IR-spectrum, ν, cm^{-1}, (KBr): 3593, 3070 (OH), 2928, 2852 (CH), 1606 (C=N), 1587 (C=C), 1510, 1444, 1433, 1294, 1190, 1146. UV (EtOH), λ_{max}nm, (lg ε): 250 (3.95), 257 (3.93), 309 (4.38), 332 (4.33), 384 (3.88). ^{1}H NMR (400 MHz, $CDCl_3$+DMSO-*d*$_6$), δ, ppm (*J*, Hz): 1.13-1.51 (10H, m, C_6H_{11}); 1.53-1.95 (10H, m, C_6H_{11}); 1.62 (6H, s, 4-CH_3); 2.86-3.04 (2H, m, Ar-C*H*-(CH_2)$_5$); 3.19 (3H, s, C*H*$_3$OH); 6.91 (2H, *AA′BB′*, *J* = 5.6, H-3′,5′ 5-ArOH); 7.97 (2H, *AA′BB′*, *J* = 5.6, H-2′,6′ 5-ArOH); 8.41 (2H, s, H-2,6 2-ArOH); 8.50 (1H, br.s, 2-ArOH); 10.16 (1H, br.s, 5-ArOH). ^{13}C NMR (100 MHz, $CDCl_3$+DMSO-*d*$_6$), δ, ppm: 24.2 (4,4-CH_3); 26.2; 26.8; 33.3 (CH_2 in C_6H_{11}); 36.7 (CH in C_6H_{11}); 49.1 (CH_3OH); 79.8 (C-4); 116.2 (C-3′,5′ 5-ArOH); 119.5 (C-1 2-ArOH); 121.9 (C-1′ 5-ArOH); 124.0 (C-2,6 2-ArOH); 129.5 (C-2′,6′ 5-ArOH); 134.2 (C-3,5 2-ArOH); 145.5 (C-2); 153.4 (C-OH 2-ArOH); 161.2 (C-OH 5-ArOH); 175.7 (C-5).

2-(3,5-Di-*tert*-butyl-4-hydroxyphenyl)-5-(4-hydroxyphenyl)-4,4-dimethyl-4*H*-imidazole 3-oxide (21s). Yellow orange powder, yield 1.67 g (82%), m.p. 285-290 °C (MeOH, dec.). Elemental analysis: found: C 73.73; H 7.95; N 6.64; calcd. for $C_{25}H_{32}N_2O_3$: C 73.50; H 7.90; N 6.86%. ^{1}H NMR (500 MHz, DMSO-d_6), δ, ppm (*J*, Hz): 1.44 (18H, s, *t*-Bu); 1.61 (6H, s, 4-CH_3); 6.94 (2H, *AA'BB'*, J_{AB} = 4.6, H-3,5 5-ArOH); 7.49 (1H, br.s, 2-ArOH); 8.01 (2H, *AA'BB'*, J_{AB} = 4.6, H-2,6 5-ArOH); 8.63 (2H, s, H-2,6 2-ArOH); 10.30 (1H, br.s, 5-ArOH). ^{13}C NMR (125 MHz, DMSO-d_6), δ, ppm: 24.1 (4-CH_3); 30.4 (C(CH_3)$_3$); 34.9 (*C*(CH_3)$_3$); 80.1 (C-4); 116.3 (C-3′,5′ 5-ArOH); 119.5 (C-1 2-ArOH); 121.9 (C-1′ 5-ArOH); 124.6 (C-2,6 2-ArOH); 129.6 (C-2′,6′ 5-ArOH); 138.7 (C-3,5 2-ArOH); 144.8 (C-2); 156.1 (C-OH 2-ArOH); 161.1 (C-OH 5-ArOH); 175.2 (C-5).

Scheme S2.

Typical reactions occurring at inhibited and non-inhibited hydrocarbon oxidation [10].

Initiation of oxidation chains:	(0) $RH \rightarrow R^{\bullet}$
Propagation of oxidation chains:	(1) $R^{\bullet} + O_2 \rightarrow RO_2^{\bullet}$<br>(2) $RO_2^{\bullet} + RH \rightarrow ROOH + R^{\bullet}$
Degenerated branching of oxidation chains:	(3) $ROOH \rightarrow RO^{\bullet} + {}^{\bullet}OH$<br>(3′) $ROOR \rightarrow RO^{\bullet} + {}^{\bullet}OR$
Termination of oxidation chains:	(4) $R^{\bullet} + R^{\bullet} \rightarrow$ R-R<br>(5) $RO_2^{\bullet} + R^{\bullet} \rightarrow$ ROOR<br>(6) $RO_2^{\bullet} + RO_2^{\bullet} \rightarrow O_2$ + molecular products<br>(7) $RO_2^{\bullet}$ + ArOH $\rightarrow$ ROOH + $ArO^{\bullet}$<br>(7′) $RO^{\bullet}$ + ArOH $\rightarrow$ ROH + $ArO^{\bullet}$<br>(7″) $R^{\bullet}$ + ArOH $\rightarrow$ RH + $ArO^{\bullet}$<br>(8) $RO_2^{\bullet} + ArO^{\bullet} \rightarrow$ molecular products

where RH – hydrocarbon (lipid); $R^{\bullet}$, $RO^{\bullet}$ and $RO_2^{\bullet}$ – alkyl, alkoxyl and peroxyl radicals, correspondingly; ROOH – organic hydroperoxide ; ArOH – radical inhibitor: $ArO^{\bullet}$ – inhibitor radical.

ESR spectra recorded for diluted and oxygen-free toluene solutions of phenoxyl-nitroxides 22a-o

(black line – experimental spectra, red line – simulated spectra). ESR parameters used for the simulations are listed in Table 3 (See part 2.3.). The presented spectra were simulated suggesting that they represent superpositions of two spectra: spectrum of HPNs **22** + spectrum of its decomposition product –nitroxide radical with unknown structure (g = 2.0059, A_N = 1.27 mT). The impurity content was suggested to be 1-5 molar percent depending on the compound.

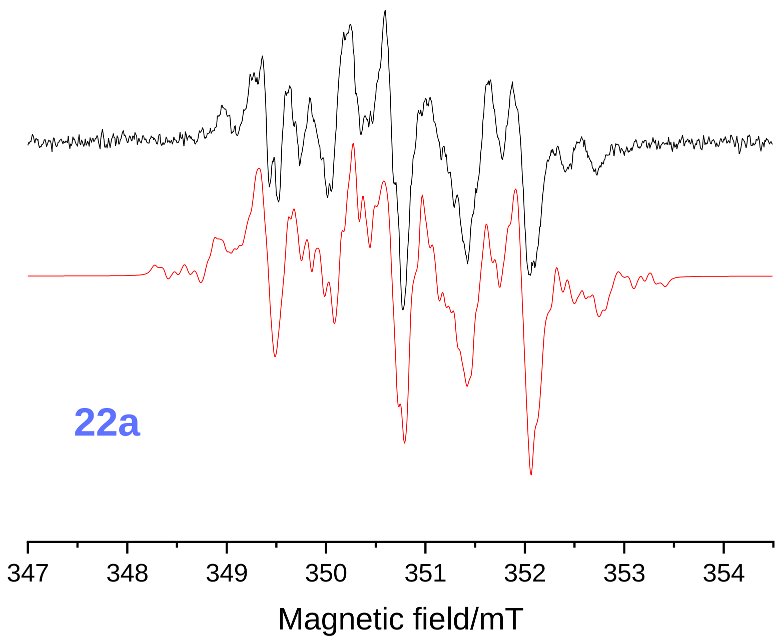

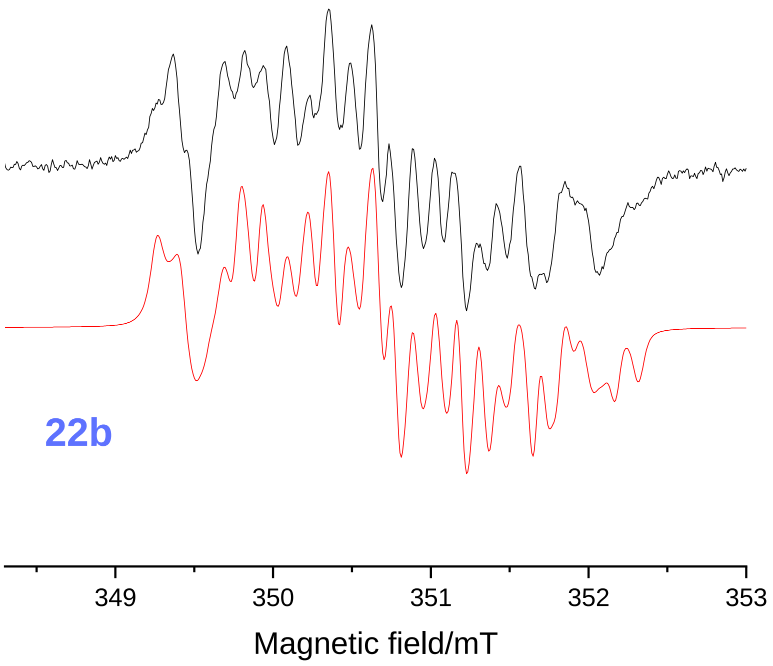

349
350
351
352
353
Magnetic field/mT

22b

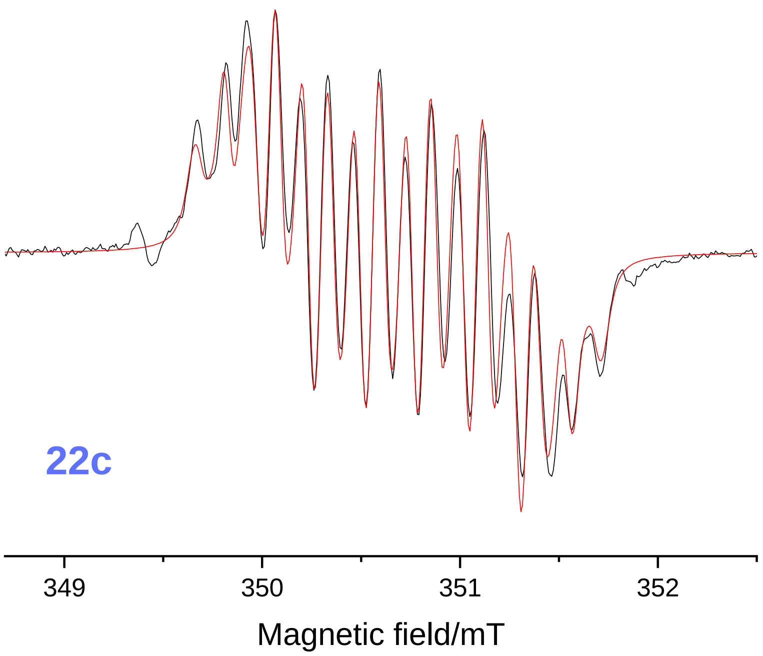

349
350
351
352
Magnetic field/mT

22c

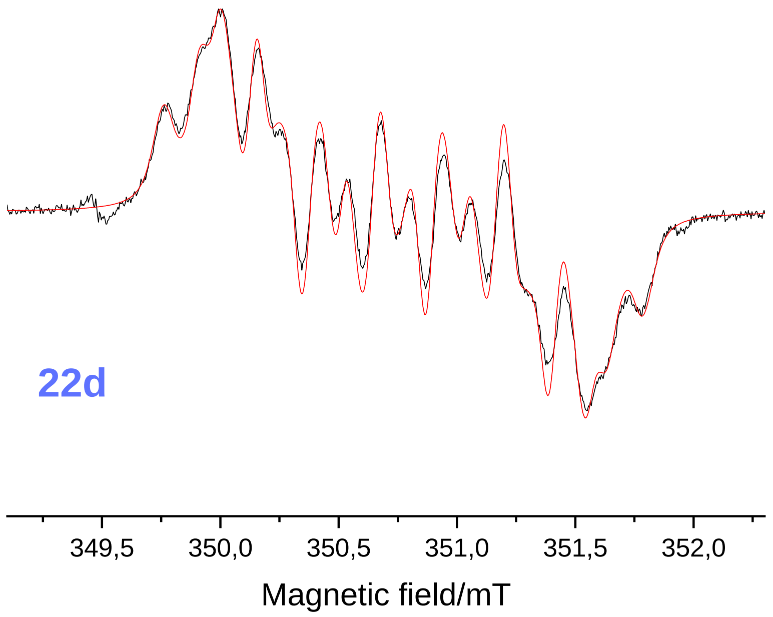
22d
349,5
350,0
350,5
351,0
351,5
352,0
Magnetic field/mT

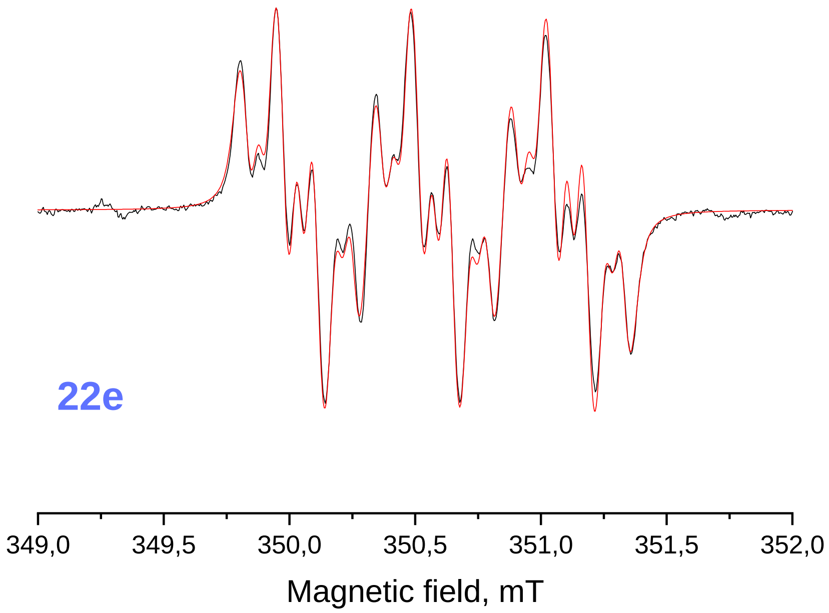
22e
349,0
349,5
350,0
350,5
351,0
351,5
352,0
Magnetic field, mT

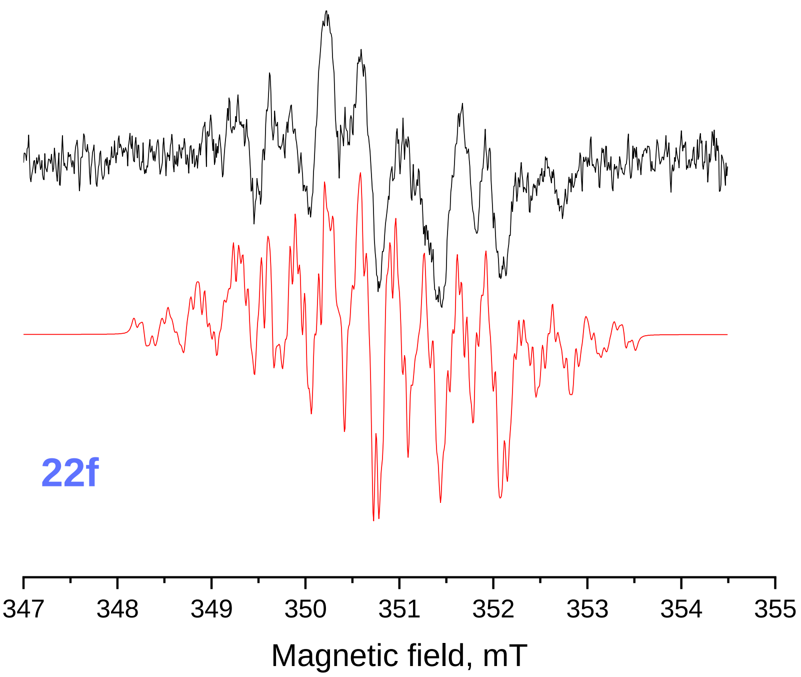
22f
347
348
349
350
351
352
353
354
355
Magnetic field, mT

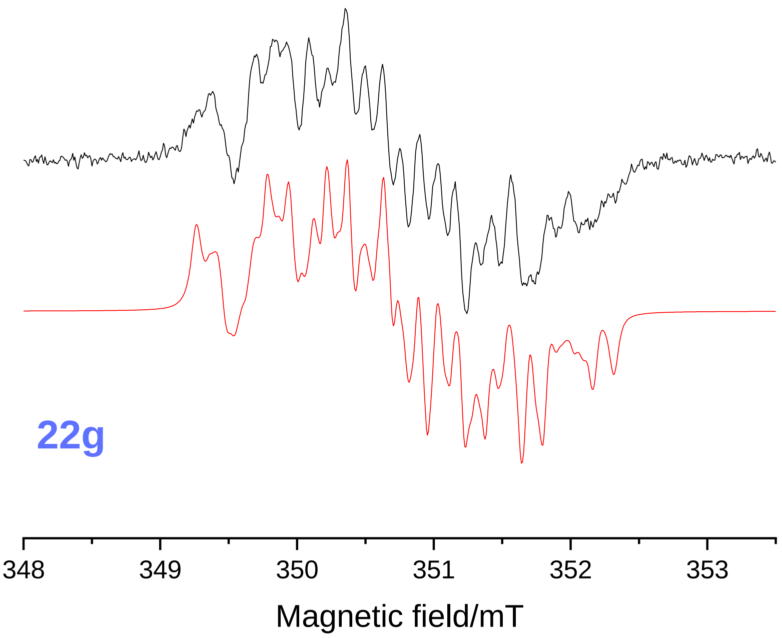
22g
348
349
350
351
352
353
Magnetic field/mT

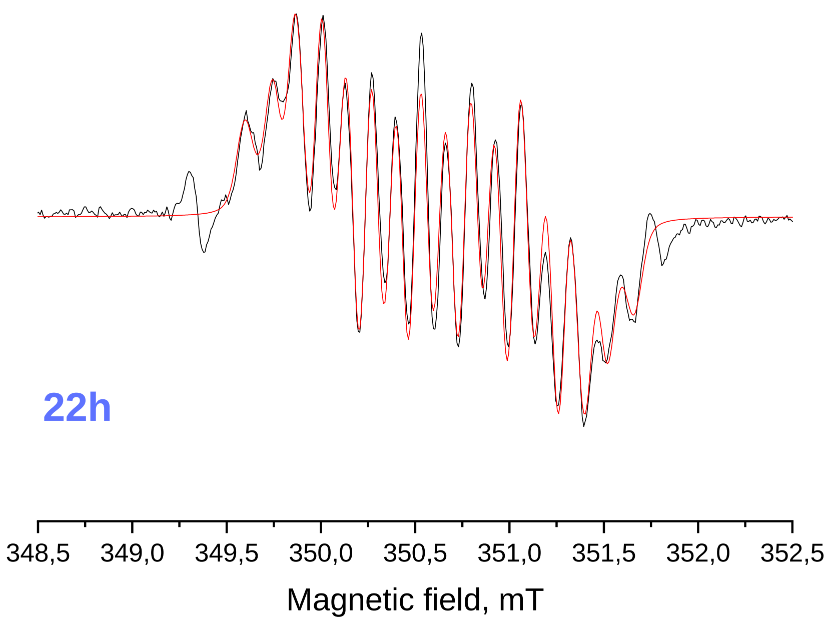
22h
348,5
349,0
349,5
350,0
350,5
351,0
351,5
352,0
352,5
Magnetic field, mT

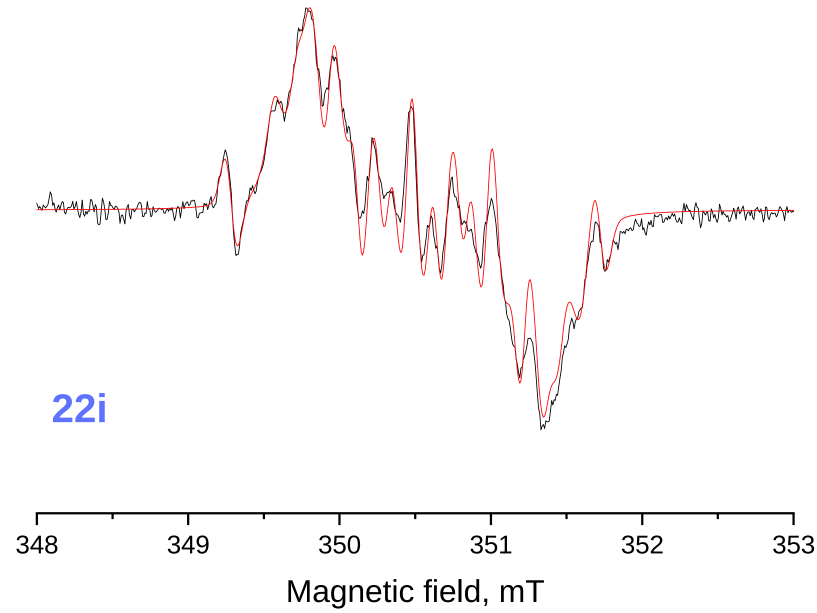
22i
348
349
350
351
352
353
Magnetic field, mT

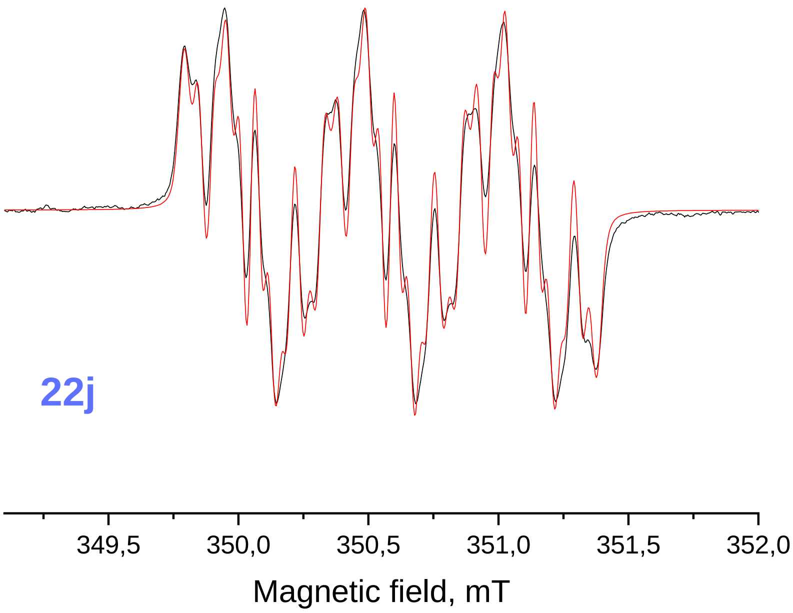
22j
349,5
350,0
350,5
351,0
351,5
352,0
Magnetic field, mT

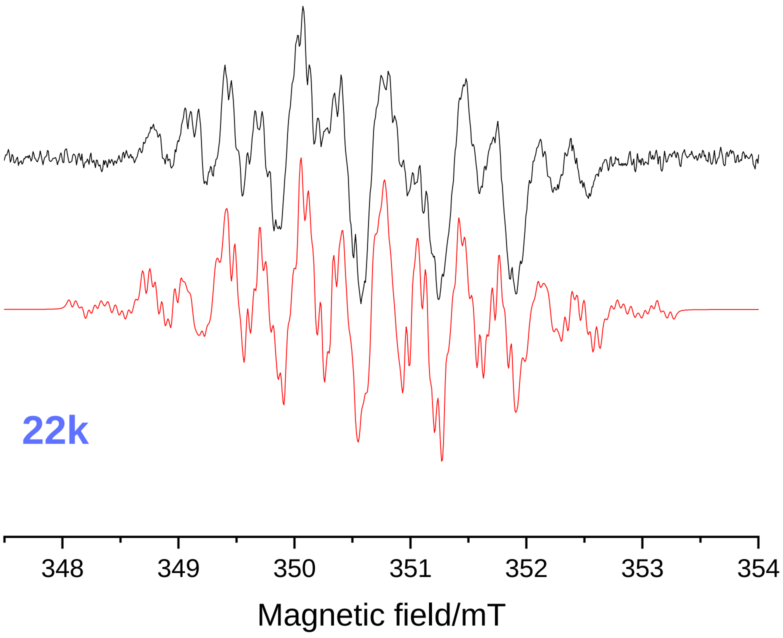
22k
348
349
350
351
352
353
354
Magnetic field/mT

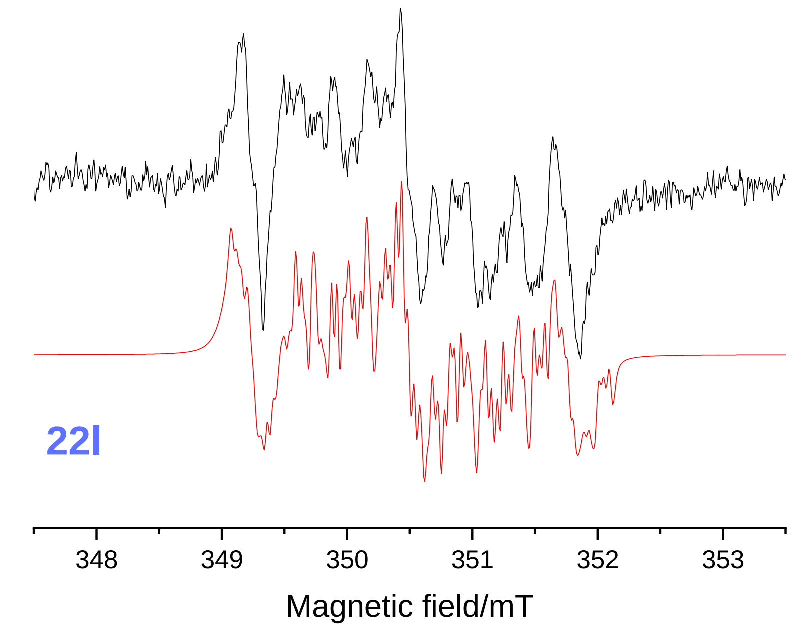
22l
348
349
350
351
352
353
Magnetic field/mT

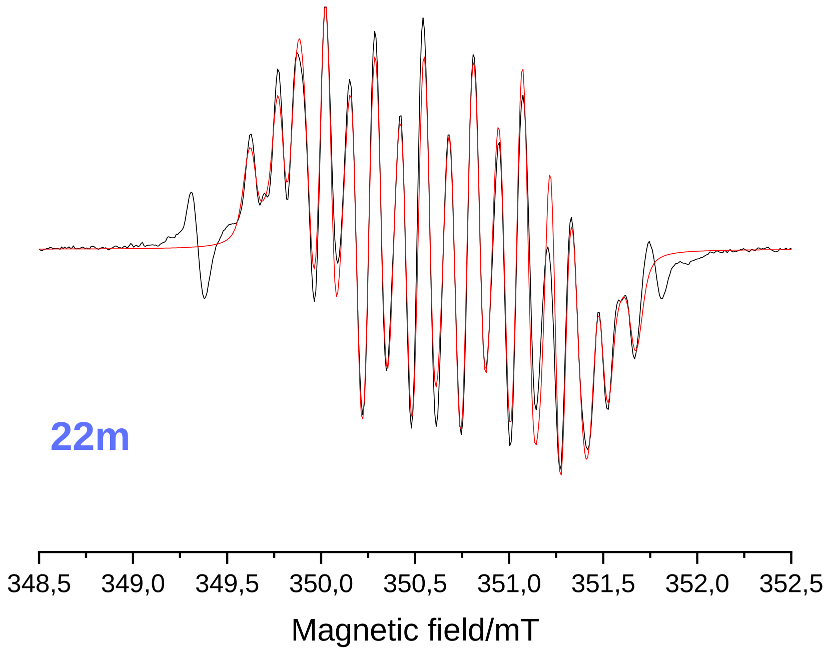
22m
348,5
349,0
349,5
350,0
350,5
351,0
351,5
352,0
352,5
Magnetic field/mT

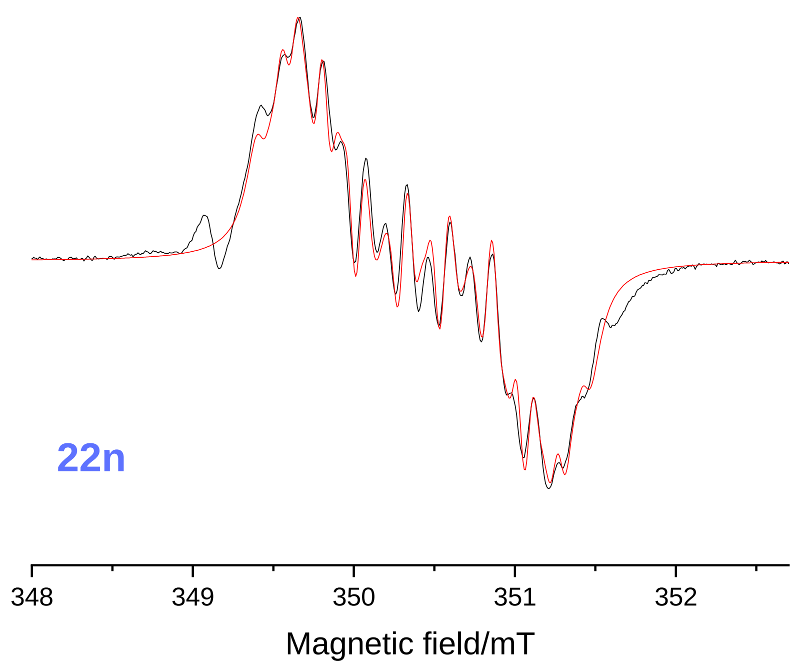
22n
348
349
350
351
352
Magnetic field/mT

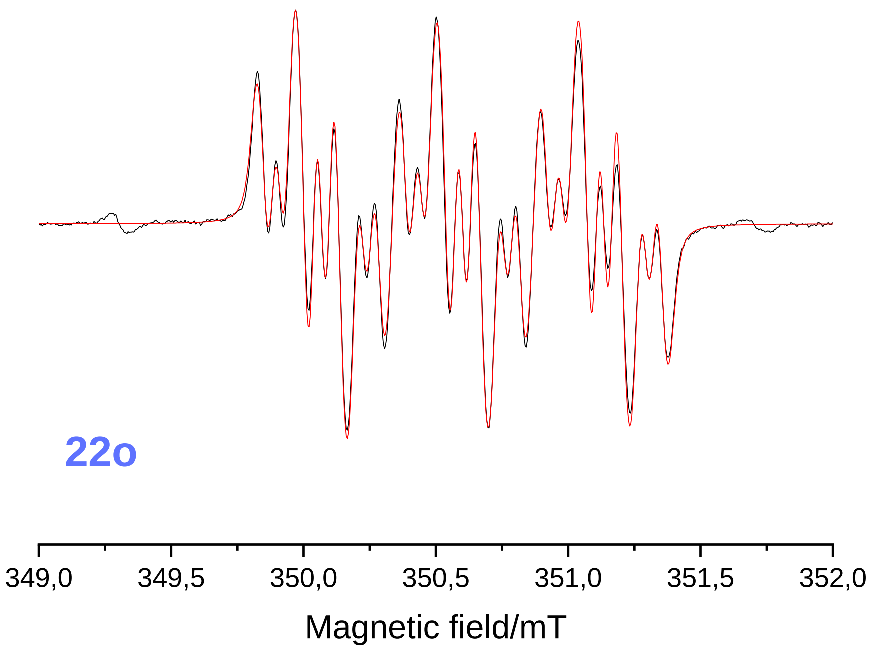
22o
349,0
349,5
350,0
350,5
351,0
351,5
352,0
Magnetic field/mT

Molecular geometry and *hfs* constants for HPNs 22a-e,j,o calculated at UB3LYP/6-31G(d) level of theory, solvent effect was taken into account using CPCM model (solvent – toluene) (Figures S1-S5).

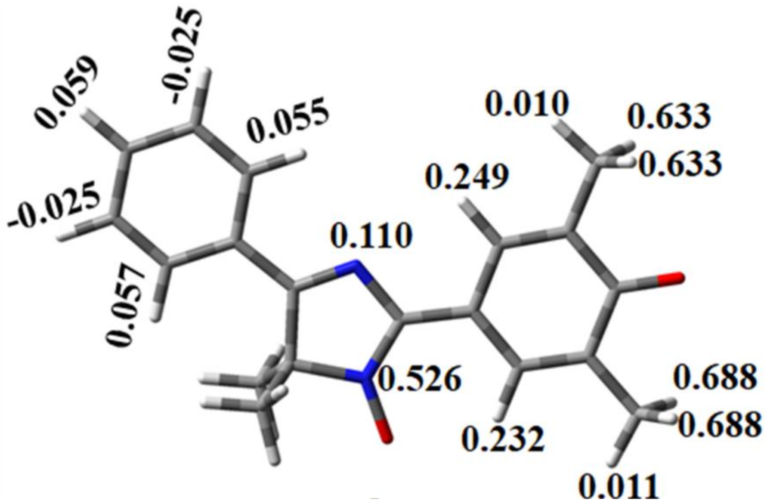

22a

Figure S1.

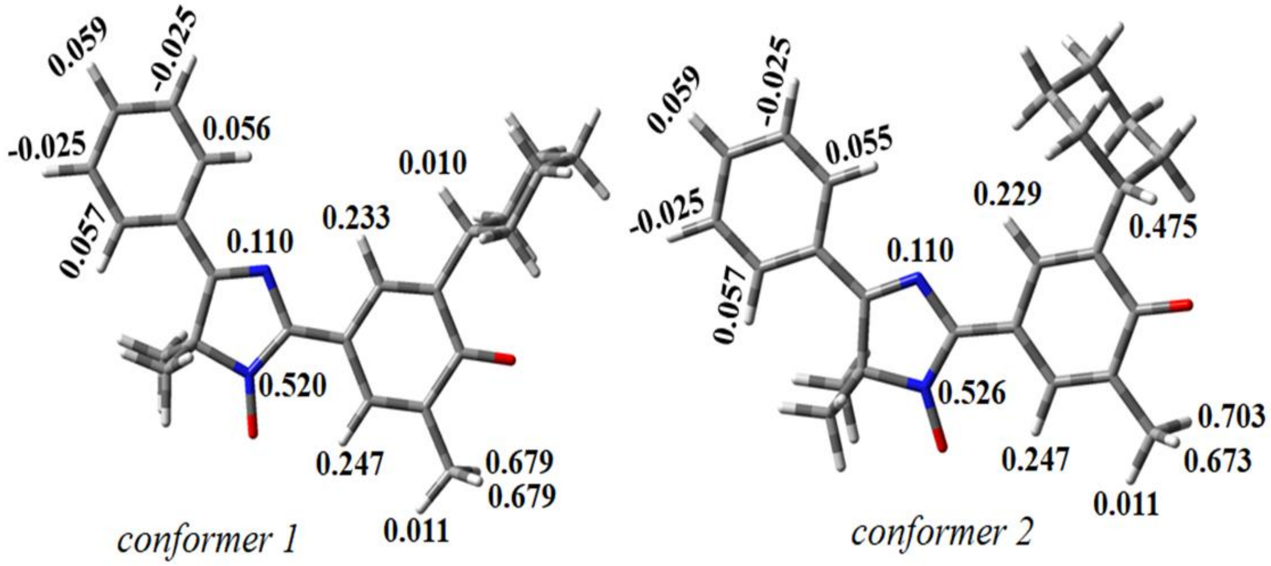

22b

Figure S2

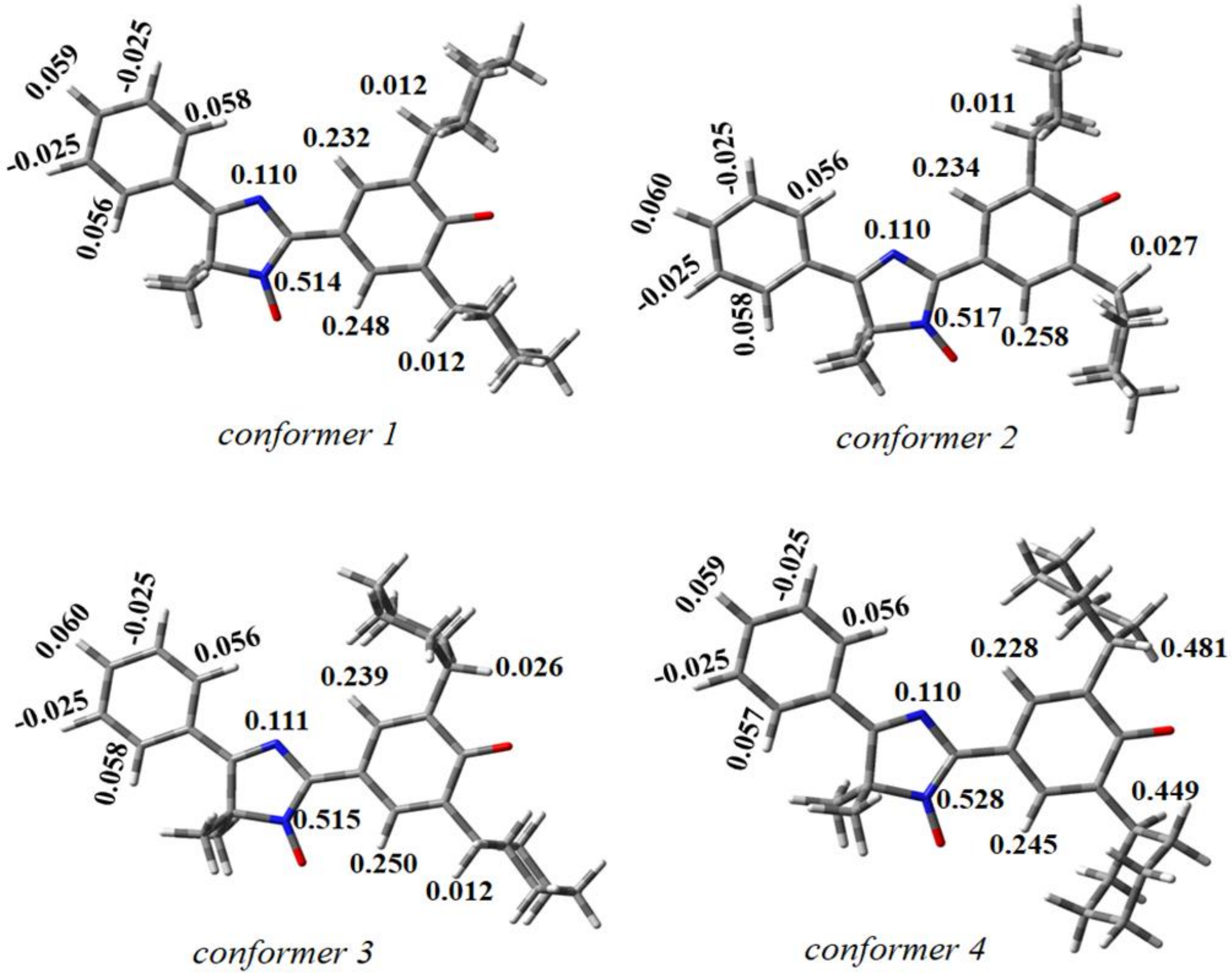

22d

Figure S3

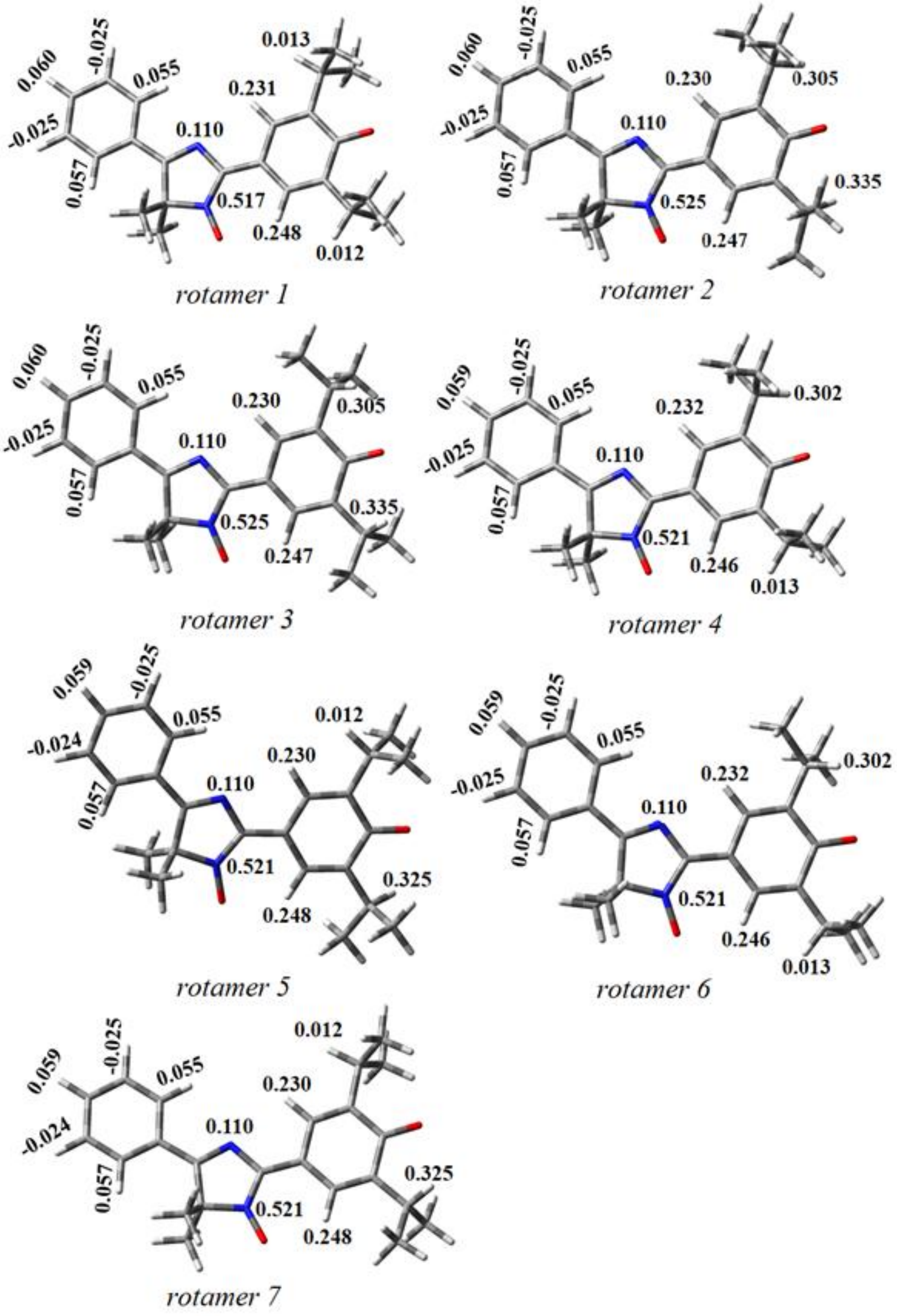

22c

Figure S4

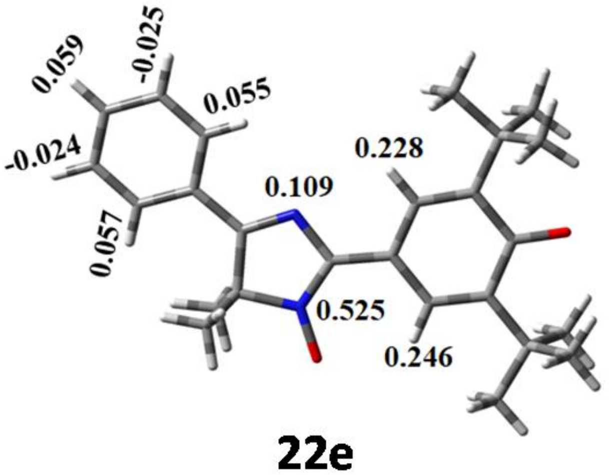

22e

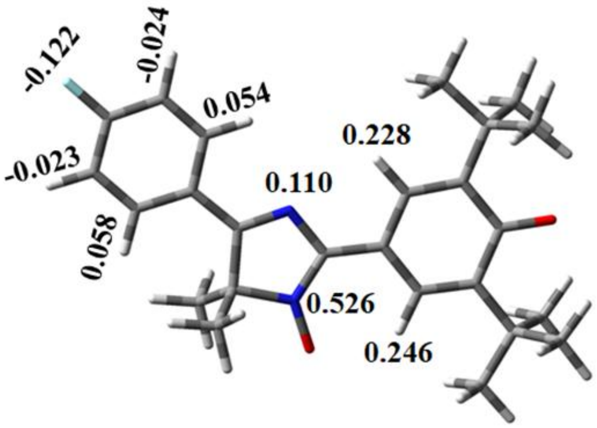

22j

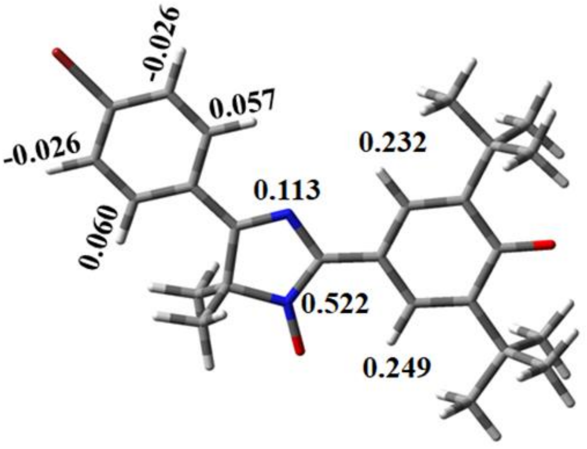

22o

Figure S5

References:

1. Rennison, D.; Conole, D.; Tingle, M.D.; Yang, J.; Eason, C.T.; Brimble, M.A. Synthesis and methemoglobinemia-inducing properties of analogues of *para*-aminopropiophenone designed as humane rodenticides. *Bioorg. Med. Chem. Lett.*, **2013**, *23*, 6629-6635. DOI: 10.1016/j.bmcl.2013.10.046
2. Colas, K.; Dos Santos, A.C.V.D.; Mendoza, A. *i*-Pr2NMgCl·LiCl Enables the Synthesis of Ketones by Direct Addition of Grignard Reagents to Carboxylate Anions. *Org. Lett.*, **2019**, *21*, 7908-7913. DOI: 10.1021/acs.orglett.9b02899
3. Quan, X.; Kerdphon, S.; Andersson, P.G. C-C Coupling of Ketones with Methanol Catalyzed by a *N*-Heterocyclic Carbene–Phosphine Iridium Complex. *Chem. Eur. J.*, **2015**, *21*, 3576–3579. DOI: 10.1002/chem.201405990
4. Frey, M.; Rohwer, H.; Wendeborn, F.; Hazenkamp, M. Metal Free Bleaching Composition. *US Pat.*, **2013**, 2013/0117941.
5. Sinhababu, A.K.; Borchardt, R.T. Selective Ring *O*-methylation of Hydroxybenzaldehydes Via Their Mannich Bases. *Synth. Commun*. **1983**, *13*, 677-683. DOI: 10.1080/00397918308060349
6. Roth, B; Baccanari, D.P.; Sigel, C.W.; Hubbell, J.P.; Eaddy, J.; Kao, J.C.; Grace, M.E.; Rauckman, B.S. 2,4-Diamino-5-benzylpyrimidines and analogs as antibacterial agents. 9. Lipophilic trimethoprim analogs as antigonococcal agents. *J. Med. Chem*. **1988**, *31*, 122-129. DOI: 10.1021/jm00396a018
7. Geurink, P.P.; Florea, B.I.; Li, N.; Witte, M.D.; Verasdonck, J.; Kuo, C.-L.; Van der Marel, G.A.; Overkleeft, H.S. A Cleavable Linker Based on the Levulinoyl Ester for Activity-Based Protein Profiling. *Angew. Chem. Int. Ed*. **2010**, *49*, 6802-6805. DOI: 10.1002/anie.201001767
8. Nikiforov, G.A.; Dyumaev, K.M.; Volod'kin, A.A.; Ershov, V.V. Inhibitors of free radical reactions. III. Formylation of 2,6-dialkylphenols. *Izv. AN USSR, Ser. Khim.* **1962**, 1836-1838; *Chem. Abstr.* **1963**, *58*, 46464.
9. Prishchenko, A.A.; Livantsov, M.V.; Novikova, O.P.; Livantsova, L.I.; Shpakovskii, D.B.; Milaeva, E.R. Addition of Trimethylsilyl Esters of Trivalent Phosphorus Acids to 3,5-Di-*tert*-butyl-4-hydroxybenzaldehyde. *Russ. J. Gen. Chem.* **2005**, *75*, 1968-1970. DOI:10.1007/s11176-006-0024-0
10. Ershov, V.V.; Nikiforov, G.A.; Volod'kin, A.A. *Sterically hindered phenols* (In Russ.); Khimiya: Moscow, USSR, 1972; pp. 1-352.
